# Neural, adipocyte and hepatic differentiation potential of primary and secondary hair follicle stem cells isolated from Arbas Cashmere goats

**DOI:** 10.1186/s12917-022-03420-3

**Published:** 2022-08-15

**Authors:** Wei Yan, Fei Hao, Xiaoshu Zhe, Yingmin Wang, Dongjun Liu

**Affiliations:** State Key Laboratory of Reproductive Regulation & Breeding of Grassland Livestock, Inner Mongolia University, Hohhot, 010021 China

**Keywords:** Hair follicle, Primary hair follicle stem cell, Secondary hair follicle stem cell, Cashmere goat, Pluripotency

## Abstract

**Background:**

Arbas Cashmere goats are excellent domestic breeds with high yields of wool and cashmere. Their wool and cashmere can bring huge benefits to the livestock industry. Our studies intend to more fully understand the biological characteristics of hair follicle stem cells (HFSCs) in order to further explore the mechanisms of wool and cashmere regular regeneration. And they have been increasingly considered as promising multipotent cells in regenerative medicine because of their capacity to self**-**renew and differentiate. However, many aspects of the specific growth characteristics and differentiation ability of HFSCs remain unknown. This study aimed to further explore the growth characteristics and pluripotency of primary hair follicle stem cells (PHFSCs) and secondary hair follicle stem cells (SHFCs).

**Results:**

We obtained PHFSCs and SHFSCs from Arbas Cashmere goats using combined isolation and purification methods. The proliferation and vitality of the two types of HFSCs, as well as the growth patterns, were examined. HFSC**-**specific markers and genes related to pluripotency, were subsequently identified. The PHFSCs and SHFSCs of Arbas Cashmere goat have a typical cobblestone morphology. Moreover, the PHFSCs and SHFSCs express HFSC surface markers, including *CD34*, *K14*, *K15*, *K19* and *LGR5*. We also identified pluripotency**-**associated gene expression, including *SOX2*, *OCT4* and *SOX9*, in PHFSCs and SHFSCs. Finally, PHFSCs and SHFSCs displayed multipotent abilities. PHFSCs and SHFSCs can be directed to differentiate into adipocyte**-**like, neural**-**like, and hepatocyte**-**like cells.

**Conclusions:**

In conclusion, this study confirmed that the biological characteristics and differentiation potential of PHFSCs and SHFSCs from Arbas Cashmere goats. These findings broaden and refine our knowledge of types and characteristics of adult stem cells.

**Supplementary Information:**

The online version contains supplementary material available at 10.1186/s12917-022-03420-3.

## Background

Mammalian hair can regenerate, a process that is regulated by the cyclic changes of morphology of the hair follicle (HF) [1]. These changes are carried out by a variety of cells, among which hair follicle stem cells (HFSCs) play a major role [2]. HFSCs are adult stem cells located in the bulge region of the HF [2]. A previous study has demonstrated that hair regeneration in nude mice can be improved by transplanting HFSCs [3]. However, when HFSCs of mice are damaged, the HF cannot initiate a new self**-**renewal cycle [4]. Hence, HFSCs are intimately associated with hair regeneration and the HF cycle.

Recently, stem cell research using domestic animals as models in veterinary science and human medicine has been extensively undertaken [5–7]. Compared with other animal models, Arbas Cashmere goat has received increasing attention, due to the wool and cashmere of Arbas Cashmere goat provided economic benefits to farms. In addition, the HF of Arbas Cashmere goat is an excellent model for studying HF regeneration. The hair growth of Arbas Cashmere goats is cyclical and annually synchronized [8], making it a more convenient model for analysis compared to other species whit asynchronous HF cycles [9, 10]. The Arbas Cashmere goat has two distinct types of HFs, namely, primary hair follicles (PHFs) that produces wool, and secondary hair follicles (SHFs) that produce the soft cashmere [11]. PHFs are larger than SHFs, and are distributed along the side of the SHF clusters in the skin [12]. PHFs and SHFs of Arbas Cashmere goats have a three**-**phase annual cycle: anagen (April**-**December), catagen (January), and telogen (February–March) [13]. The proliferation rate of HF cells decreases from the anagen to catagen phase [14]. In the catagen phase, the hair cells begin apoptosis; simultaneously, the HF rapidly retrogresses and shortens until the hair bulb at the bottom of the HF is adjacent to the bulge region [15]. Subsequently, most HFs enter the telogen phase. Quiescent HFSCs are activated by dermal papilla cells (DPCs), located in the hair bulb of each HF, to proliferate and differentiate. The new HF then begins to form during the telogen to anagen phase [16]. However, relatively few studies have examined the mechanism of HF regeneration in goats, including the function of HFSCs.

HFSCs are multipotent and, as such, play significant roles in hair regeneration. This multipotent capacity can also be exploited in tissue engineering. For example, HFSCs can maintain skin homeostasis and repair wounds after skin injury [17]. Several studies have also demonstrated the potential of human and mouse HFSC application in tissue engineering, including that of bones, blood vessels, and nerves, etc. [18–20]. Meanwhile, the multipotency of primary hair follicle stem cells (PHFSCs) and secondary hair follicle stem cells (SHFSCs) of the Arbas Cashmere goat are not yet fully understood, thus, this lack of fundamental knowledge about PHFSCs and SHFSCs hinder their application in tissue engineering.

Various surface markers can be used to identify PHFSCs and SHFCs. The most common surface markers of HFSCs are *K19*, *K15*, *CD34*, *LGR5* and *K14.* They are found to be the most abundantly expressed marker in mice HFSCs [21, 22]. Meanwhile, *CD34* and *K15* are co-localised in the bulge region of mouse and human HFs [22, 23]. Furthermore, *LGR5* is also a maker of HFSCs [24]. K14 + cells are located within the HF bulge region and the basal layer of the epidermis [25].

To determine the multipotency of PHFSCs and SHFSCs, we quantify the expression of core pluripotency factors, including *SOX9*, *OCT4* and *SOX2* [26]. *SOX9* is a factor involved in HF development, including hair differentiation and maintenance of pluripotency [27]. In addition, we can induce differentiation of PHFSCs and SHFSCs into adipocyte-like, neural-like cells, and hepatocyte-like cells and evaluate the degree of differentiation by related gene expression. For example, *NeuN* have an essential function in the nerve-formation process [28]. Meanwhile, *GFAP* [29], and *MAP2* [30] genes are commonly used as a particular marker for nerve cells. *PPARG* and *FABP4* make key contributions to adipogenesis and promote fat accumulation [31]. *ADIPOQ* [32] and *Leptin* [33] are two factors secreted by adipocytes. *AFP* [34] and *ALB* [35] are secreted by the liver, While *HNF4A* [36] acts as a transcriptional factor to maintain hepatic function.

In this study, PHFSCs and SHFSCs were isolated from Arbas Cashmere goats to elucidate their growth and multipotent differentiation characteristics. In so doning, we aimed to provide an experimental foundation for future functional research on HFSCs and HF cycle mechanisms. This theoretical foundation also has remarkable significance for the HFSCs application in the tissue regeneration and stem cell therapy.

## Results

### Isolation and culture of HFSCs

**Fig. 1:**
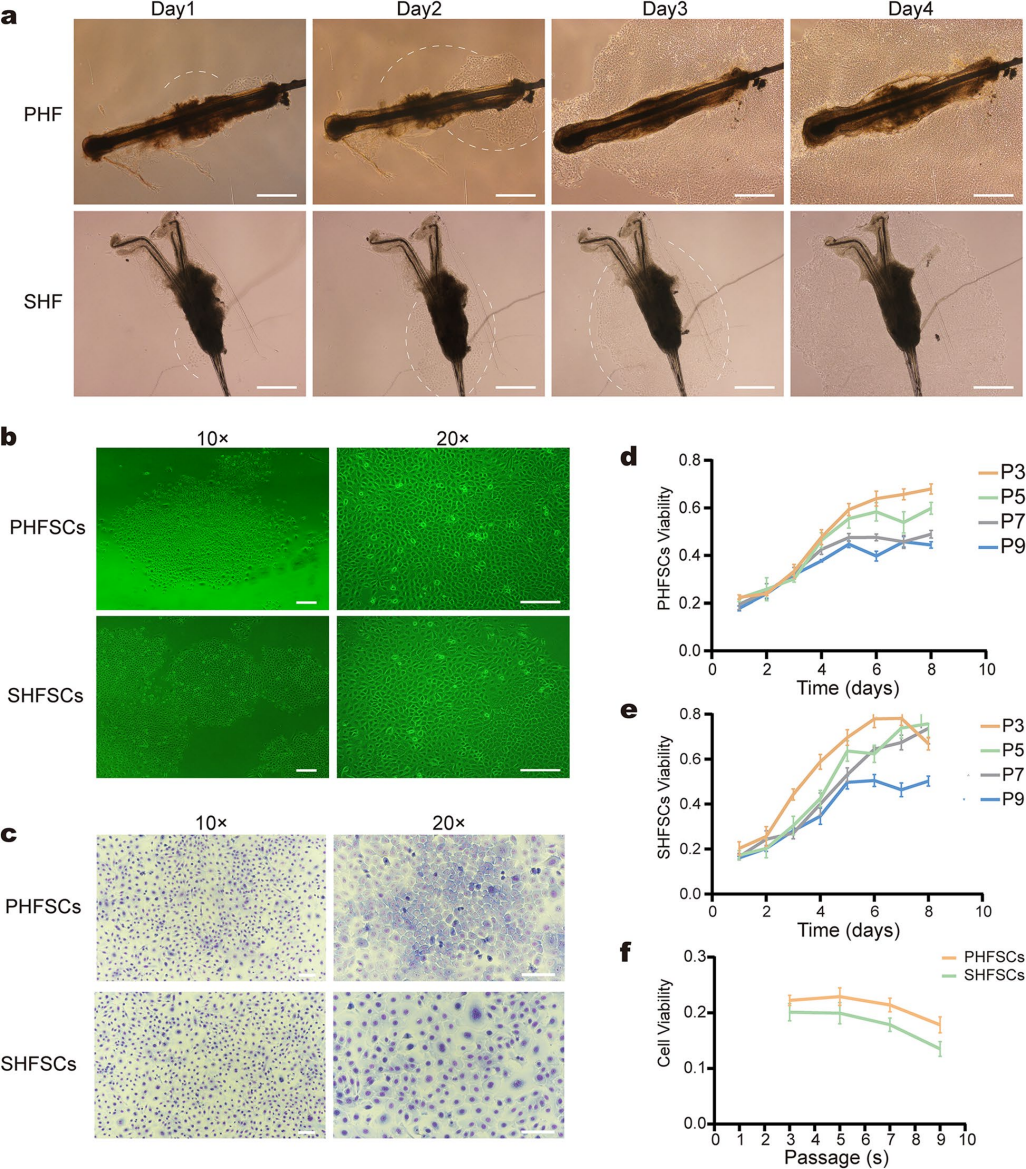
Isolation and culture of HFSCs. **a** Primary hair follicles (PHFs) and secondary hair follicles (SHFs) isolated from the skin of Arbas Cashmere goats. The dotted lines represent the migrated cells following culture for 1–4 days (magnification × 4; Scale bar, 500 µm). **b** Primary hair follicle (PHF) stem cells (PHFSCs) and secondary hair follicle (SHF) stem cells (SHFCs) purified using type IV collagen (magnification × 10 and × 20; scale bar, 100 µm). **c** PHFSCs and SHFSCs examined under a microscope following Giemsa staining (magnification × 10 and × 20; scale bar, 100 µm). **d** Growth curves of PHFSCs at the third (P3), fifth (P5), seventh (P7), and ninth (P9) passages. **e** Growth curves of SHFSCs at P3, P5, P7, and P9. **f** Viability of PHFSCs and SHFSCs at P3, P5, P7, and P9

All PHFs were longer and larger diameters than the SHFs. Moreover, the profile of PHF hair bulb was clearer than that of SHFs. A few cells migrated from around the HF bulge region after 3–4 days of culture and the cells migrated more rapidly on days 4–5 (Fig. 1a).

Both PHFSCs and SHFSCs grow in a tightly arranged, cobblestone**-**like morphology, which is typical for skin stem cells [37]. HFSCs were small and polygonal, with a high refractive index (Fig. 1b). The cells contained multiple nuclei and resembled a nest (Fig. 1c). PHFSCs and SHFSCs had similar morphology and size; no evident differences were observed using the naked eye.

### HFSC viability and proliferation

The viability and proliferation of HFSCs were determined based on the CCK-8 assay. The HFSC growth curve was s-shaped; cells proliferated slowly over days 1–3 and then rapidly when they entered the logarithmic growth phase. Moreover, the proliferation rate and viability of the two cell types gradually decreased with increasing passages (Fig. 1d–e). At the same passages, the cell viability of PHFSCs was higher than that of SHFSCs (Fig. 1f). These results suggested that 3rd or 5th passage cells, whether PHFSCs or SHFSCs, were optimal for subsequent experiments.

### Identification of HFSCs

**Fig. 2:**
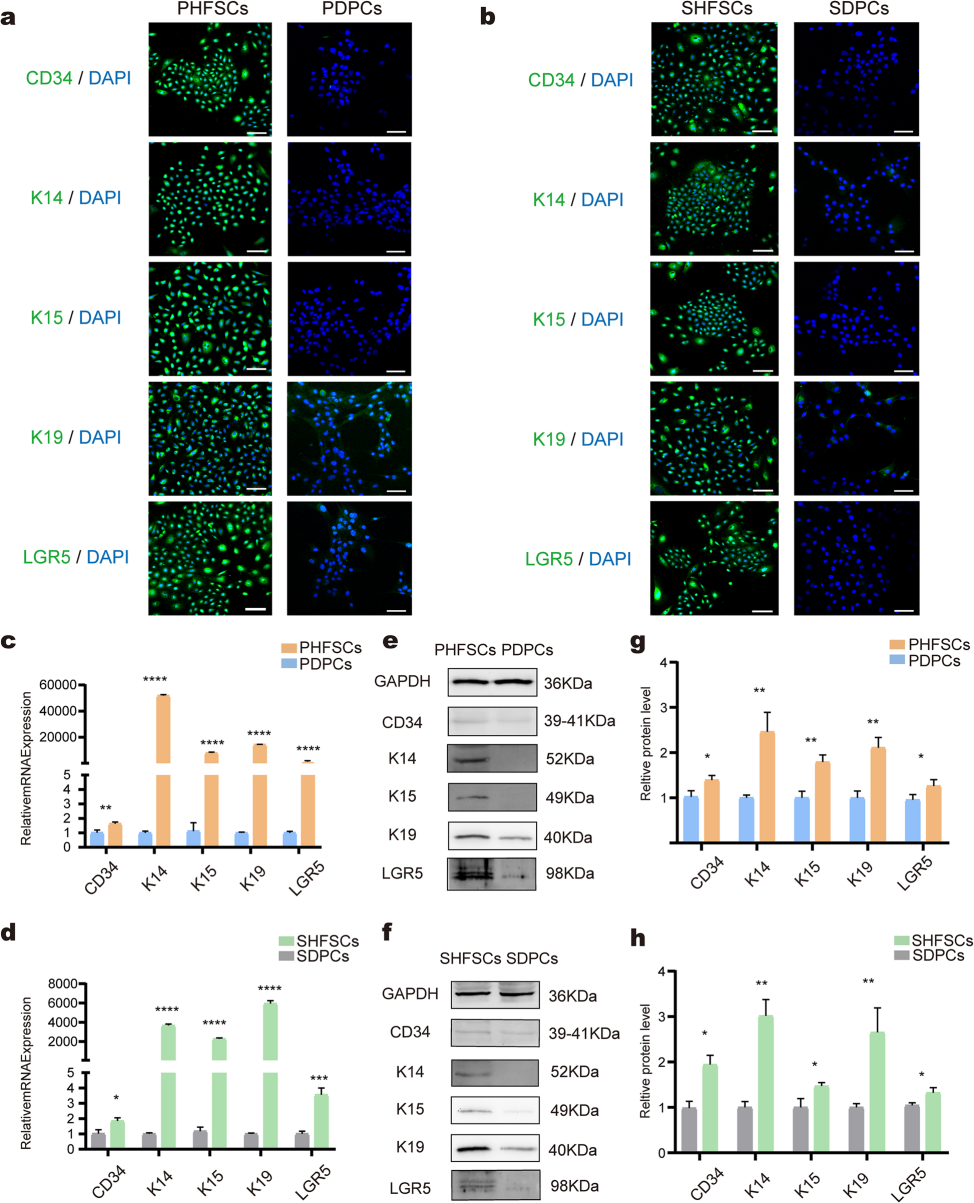
Identification of HFSCs. **a** Immunofluorescence to check for the expression of CD34, K14, K15, K19 and LGR5 in PHFSCs; nuclei (blue), target proteins (green) (Scale bar, 100 µm). **b** Immunofluorescence to check for the expression of CD34, K14, K15, K19 and LGR5 in SHFSCs; nuclei (blue), target proteins (green) (Scale bar, 100 µm). **c** Transcript-level expression of *CD34*, *K14*, *K15*, *K19*, and *LGR5* in PHFSCs. PDPCs were used as a control. **d** Transcript-level expression of *CD34*, *K14*, *K15*, *K19*, and *LGR5* in SHFSCs. SDPCs were used as a control. **e** Western blotting to check the expression of CD34, K14, K15, K19, and LGR5 in PHFSCs. Full-length blots are presented in Supplementary Fig. 1. **f** Western blotting to check the expression of CD34, K14, K15, K19, and LGR5 in SHFSCs. Full-length blots are presented in Supplementary Fig. 1. **g** Grey-value quantitative analysis of CD34, K14, K15, K19, and LGR5 in PHFSCs. PDPCs were used as a control. **h** Grey-value quantitative analysis of CD34, K14, K15, K19, and LGR5 in SHFSCs. SDPCs were used as a control. * *P* < 0.05, ** *P* < 0.01, *** *P* < 0.001, **** *P* < 0.0001

Immunofluorescence analysis showed that the PHFSCs were *CD34*, *K14*, *K15*, *K19* and *LGR5* positive, similar to SHFSCs (Fig. 2a–2b). Compared with the PHFSCs and SHFSCs, little or no green fluorescent signal could be detected in the control groups (PDPCs and SDPCs). *K14*, *K15*, *K19*, and *LGR5* were expressed at significantly higher levels in PHFSCs when compared to the control group. *CD34* expression was 1.6-fold higher than the control group, indicating that the markers were expressed at moderate levels in PHFSCs (Fig. 2c). Similarly, *K14*, *K15*, *K19*, and *LGR5* gene expression were significantly higher in SHFSCs, compared to the control group. The expression of *CD34* in SHFSCs was 1.8-fold higher than that in SDPCs, indicating that *CD34* was expressed at moderate levels in SHFSCs. (Fig. 2d).

Western blotting revealed that *CD34*, *K14*, *K15*, *K19*, and *LGR5* were expressed at high levels in PHFSCs and SHFSCs compared to control PDPCs and SDPCs respectively (Fig. 2e–f). Furthermore, the results corresponded to those of Grey-value quantitation (Fig. 2g–h).

### Pluripotency of HFSCs

**Fig. 3:**
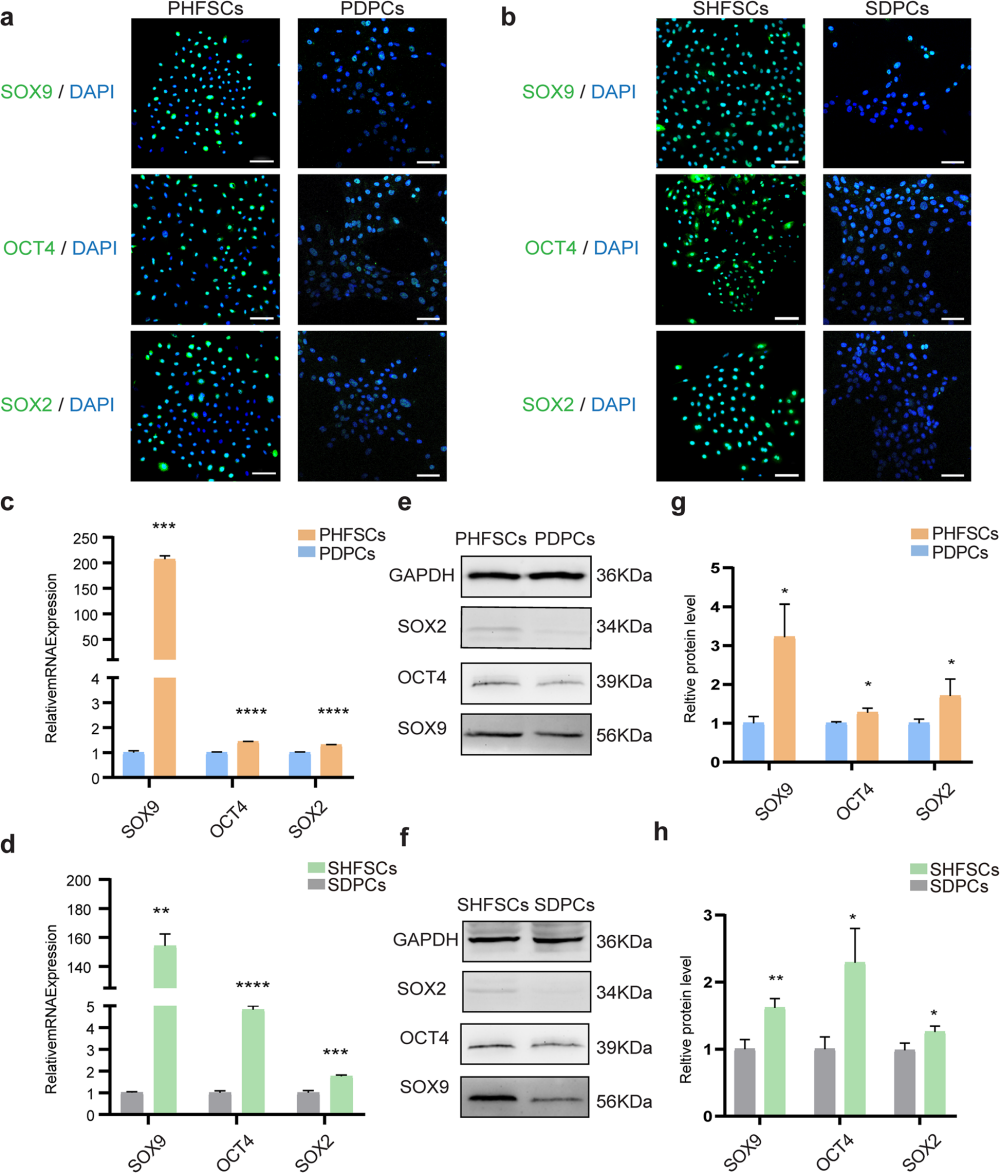
Expression of pluripotency-related genes in HFSCs. **a** Immunofluorescence was used to check the expression of SOX9, OCT4, and SOX2 in PHFSCs; nuclei (blue) and target proteins (green) (Scale bar, 100 µm). **b** Detection of SOX9, OCT4, and SOX2 expression in SHFSCs by the immunofluorescence (Scale bar, 100 µm). **c** qRT-PCR was used to evaluate the transcript-level expression of *SOX9*, *OCT4*, and *SOX2* in PHFSCs. PDPCs were used as a control. **d** qRT-PCR was used to evaluate the transcript-level expression of *SOX9*, *OCT4*, and *SOX2* in SHFSCs. SDPCs were used as a control. **e** Western blotting was used to check the expression of SOX9, OCT4, and SOX2 in PHFSCs and PDPCs. Full-length blots are presented in Supplementary Fig. 1. **f** Western blotting was used to check the expression of SOX9, OCT4, and SOX2 was assessed in SHFSCs and SDPCs. Full-length blots are presented in Supplementary Fig. 1. **g** Grey-value quantitative analysis of SOX9, OCT4, and SOX2 expression in PHFSCs. PDPCs were used as a control. **h** Grey-value quantitative analysis of SOX9, OCT4, and SOX2 expression in SHFSCs. SDPCs were used as a control. * *P* < 0.05, ** *P* < 0.01, *** *P* < 0.001, **** *P* < 0.0001

The expression of pluripotency factors *SOX2*, *SOX9*, and *OCT4* was used to identify PHFSCs and SHFSCs via immunofluorescence. Results reveal positive *SOX2*, *SOX9*, and *OCT4* expression in PHFSCs and SHFSCs (Fig. 3a–b). Compared with the PHFSCs and SHFSCs, little or no green fluorescent signal could be detected in the control groups (PDPCs and SDPCs).

qRT-PCR analysis further revealed that *SOX9, SOX2* and *OCT4* had higher expression in PHFSCs than the control group PDPCs (Fig. 3c). As shown in Fig. 3d, higher mRNA expression levels of *SOX9*, *OCT4*, and *SOX2* were observed in SHFSCs compared with control group SDPCs (Fig. 3d).

Furthermore, the abundance of SOX9, OCT4 and SOX2 protein in PHFSCs and SHFSCs were confirmed by western blotting, revealing markedly higher SOX9, OCT4 and SOX2 abundance in the PHFSCs and SHFSCs compared with that in the control groups (Fig. 3e–f). These results were consistent with those of Grey-value quantitation (Fig. 3g–h). Collectively, these results indicated that pluripotent genes were highly expressed in HFSCs.

### Multidirectional differentiation ability of HFSCs

We further verified the multipotency of HFSCs from Arbas Cashmere goats by inducing HFSCs to differentiate into neurocytes, adipocytes and liver-like cells.

### Neurogenic differentiation of HFSCs

**Fig. 4:**
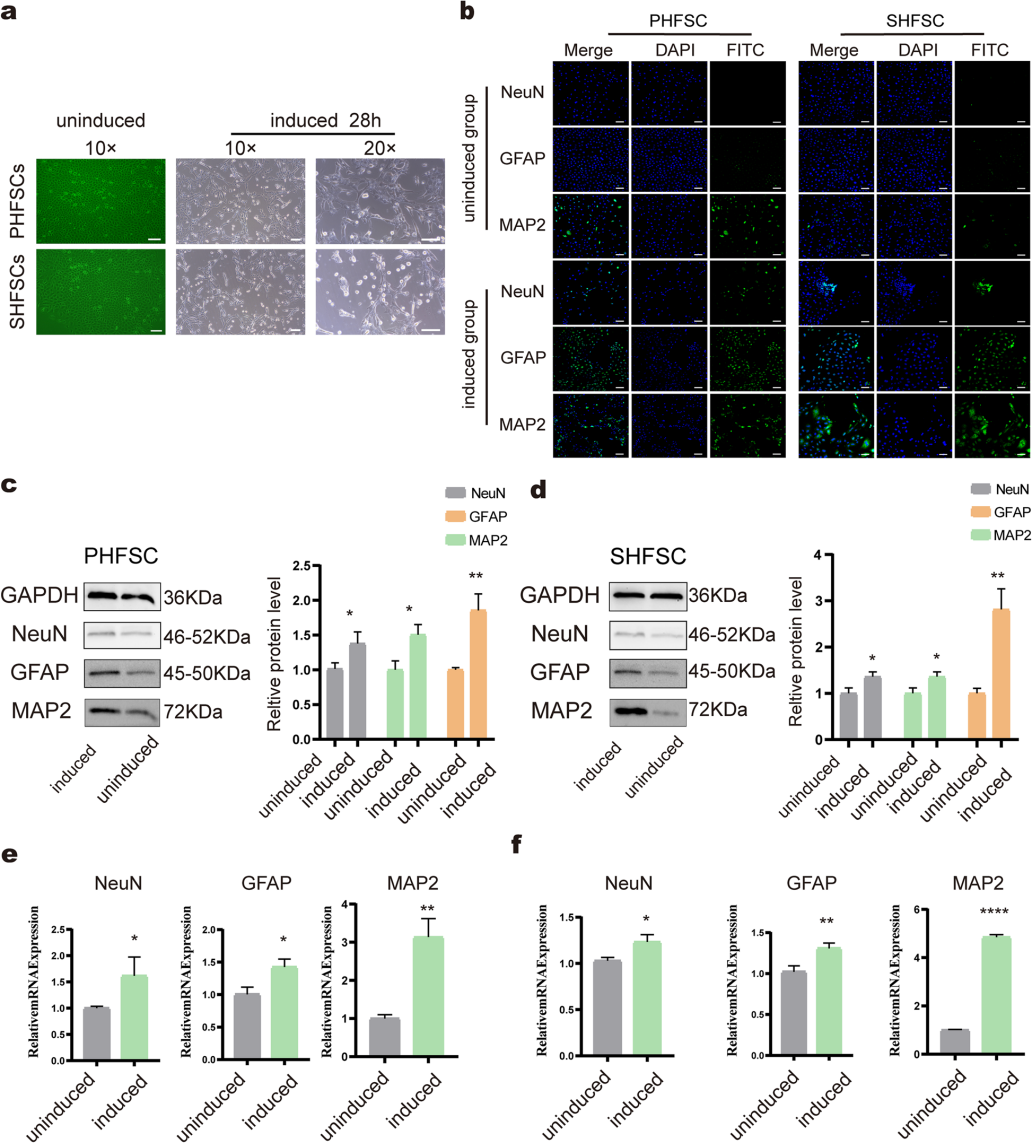
Neurogenic differentiation of HFSCs. **a** The first column represents the uninduced group of PHFSCs and SHFSCs (magnification × 10; scale bar, 100 µm). The second and third columns represent the differentiation of PHFSCs and SHFSCs into nerve cells (magnification × 10 and × 20; scale bar, 100 µm). **b** The expression of NeuN, GFAP, and MAP2 in PHFSCs and SHFSCs were determined using immunofluorescence; nuclei (blue), target proteins (green). **c** Western blotting to check the expression of NeuN, GFAP, and MAP2 in PHFSCs and Grey-value quantitative analysis. Full-length blots are presented in Supplementary Fig. 2. **d** Western blotting to check the expression of NeuN, GFAP, and MAP2 in SHFSCs and Grey-value quantitative analysis. Full-length blots are presented in Supplementary Fig. 2. **e** Transcript-level expression of *NeuN*, *GFAP*, and *MAP2* in the induced PHFSC group. The control group comprised the uninduced PHFSCs. **f** Transcript-level expression of *NeuN*, *GFAP*, and *MAP2* in the induced SHFSCs. The control group comprised the uninduced SHFSCs (Scale bars, 100 µm). * *P* < 0.05, ** *P* < 0.01, *** *P* < 0.001

The morphological characteristics of HFSCs were assessed after induction of differentiation. Under normal culture conditions, the untreated PHFSCs and SHFSCs exhibited a cobblestone-like morphology (Fig. 4a), however, morphological changes were observed in the PHFSCs and SHFSCs after treatment 28 h with the induction medium. Specifically, neuron-like cells appeared with the cytoplasm of both types of HFSCs gathered towards the nucleus. Additionally, most HFSCs presented a high refractive index, and the cells formed network-like structures with several branches or junctions. representing bipolar or multipolar cells. PHFSC and SHFSC differentiate into neural-like cells with bipolar or multipolar morphology( Fig. 4a).

As revealed via immunofluorescent staining, NeuN was weakly expressed in the induced PHFSCsgroup, however, not in the uninduced group. Compared with the uninduced group, the number of cells expressing GFAP in the induced group increased. The expression of MAP2 was detected before and after differentiation of PHFSCs (Fig. 4b). In uninduced SHFSCs, the expression of NeuN was negligible, while a faint fluorescence signal was detected in induced SHFSCs. Both GFAP and MAP2 were expressed in a few uninduced SHFSCs, however, the number of cells expressing GFAP and MAP2 increased compared to that observed before induction (Fig. 4b). These results indicated that the PHFSCs and SHFSCs began to differentiate into nerve cells.

After 28 h of induction, western blotting revealed that NeuN, GFAP, MAP2 were expressed at high levels in PHFSCs and SHFSCs induced group compared to uninduced PHFSCs and SHFSCs respectively (Fig. 4c–d). Furthermore, the results corresponded to those of Grey-value quantitation (Fig. 4c–d). qRT-PCR analysis further revealed that *NeuN, GFAP, MAP2* had higher expression in PHFSCs and SHFSCs induced group than uninduced PHFSCs and SHFSCs respectively (Fig. 4e–f).

### Adipogenic differentiation of HFSCs

**Fig. 5:**
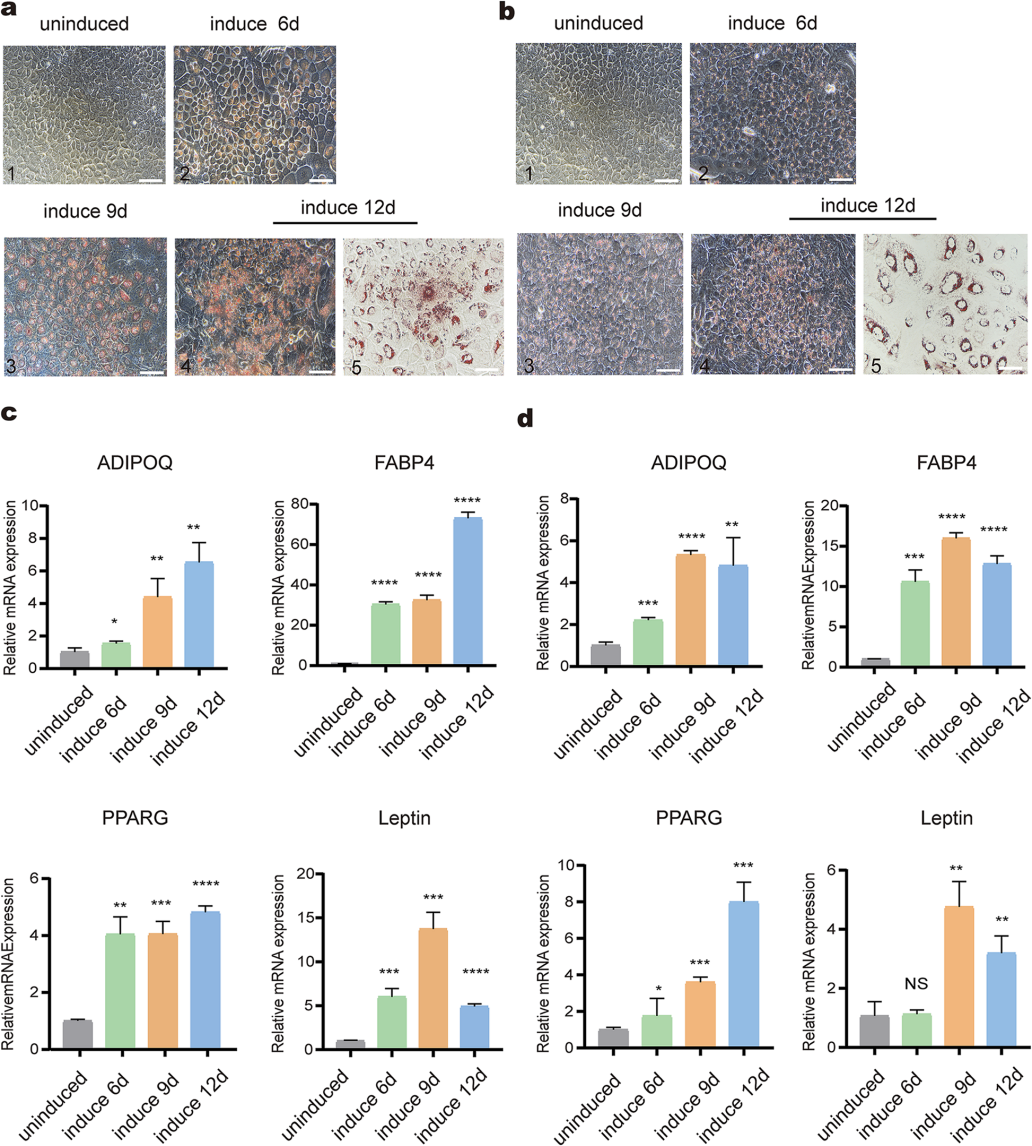
Adipogenic differentiation of HFSCs. **a** Oil Red O staining was used to evaluate the formation of lipid droplets on days 6, 9, and 12 after the differentiation of PHFSCs into adipocytes (Scale bar: 1–4, 100 µm; 5, 50 µm). **b** The formation of lipid droplets was detected by Oil Red O stainingon days 6, 9, and 12 after the differentiation of SHFSCs into adipocytes (Scale bar: 1–4, 100 µm; 5, 50 µm). **c** Transcript-level expression of *ADIPOQ*, *FABP4*, *PPARG*, and *Leptin* in the induced PHFSCs, with a control group of uninduced PHFSCs. **d** Transcript-level expression of *ADIPOQ*, *FABP4*, *PPARG*, and *Leptin* in the induced SHFSCs, with a control group of uninduced SHFSCs. * *P* < 0.05, ** *P* < 0.01, *** *P* < 0.001, **** *P* < 0.0001; NS, not significant

We treated PHFSCs and SHFSCs with an induced medium for 12 days. Oil droplets formed, and the degree of differentiation into adipocytes was proved using Oil Red O staining. Following inducing differentiation for 6 days, a few bright orange lipid droplets were observed around the margins of the nuclei in PHFSCs and SHFSCs. As the induction time increased, the number of lipid droplets secreted by the two types of HFSCs gradually increased, and small red lipid droplets were secreted into the extracellular space. Following inducing differentiation for 15 days, small polygonal lipid droplets gradually fused to form larger and round lipid droplets. The droplets secreted by the PHFSCs were smaller and more numerous than the SHFSCs. No lipid droplets were observed in the uninduced PHFSCs and SHFSCs with normal culture(Fig. 5a–b).

In addition, the total RNA of induced PHFSCs and SHFSCs were collected separately at three different time points (days 6, 9, and 12) and used to analyse the mRNA levels of adipogenic differentiation-related genes. When PHFSCs were induced to differentiate into adipocytes, the mRNA expression of *ADIPOQ*, *Leptin*, *FABP4*, and *PPARG* increased (Fig. 5c). Similarly, the mRNA expression of *ADIPOQ*, *PPARG*, *Leptin* and *FABP4* were increased in SHFSCs induced to differentiate into adipocytesn (Fig. 5d).

### Differentiation of HFSCs into liver-like cells

**Fig. 6:**
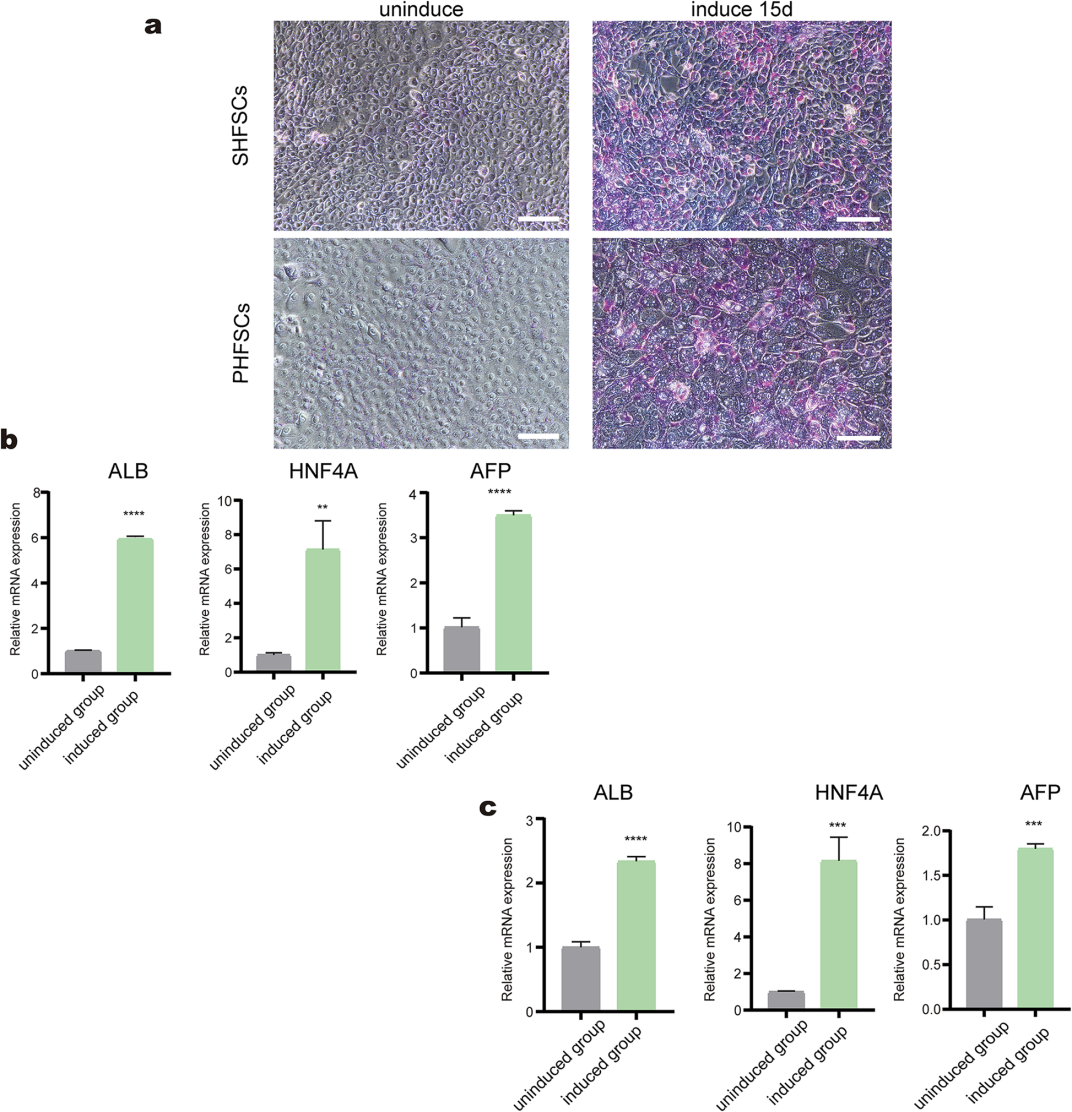
Hepatic differentiation of HFSCs (**a**) Glycogen was detected by glycogen staining (PAS). Compared with the control group, most cells were purplish red, indicating positive PAS staining, and the nuclei were stained blue-purple (scale bar, 100 µm). **b** Transcript-level expression of *ALB*, *HNF4A*, and *AFP* in the induced PHFSCs, with a control group of uninduced PFHSCs. **c** Transcript-level expression of *ALB*, *HNF4A*, and *AFP* in the induced SHFSCs, with a control group of uninduced SHFSCs. ** *P* < 0.01, *** *P* < 0.001, **** *P* < 0.0001

During the early induction phase, PHFSCs and SHFSCs growth were inhibited, however, the cells growth recovered at later time points. As induction time increased, the cells volume of PHFSCs and SHFSCs also increased, while the size of the nucleus decreased. The nucleus from PHFSCs and SHFSCs became more prominent, with a few vesicles appearing around the nucleus, and the cells attained an irregularly round or triangular shape (Fig. 6a). After 14 days of induction, glycogen was detected using PAS staining. Glycogen levels increased in the induced group, and the nuclei stained blue in the purplish-red cytoplasm (Fig. 6a).

qRT-PCR was used to evaluate the expression of *ALB*, *HNF4A*, and *AFP* to demonstrate differentiation of HFSCs into hepatocytes. In the induced PHFSCs, the mRNA expression of *ALB*, *HNF4A*, and *AFP* was 5.9-, 8.1-, and 3.5-fold higher than that in the uninduced PHFSCs group, respectively (Fig. 6b). In the induced SHFSCs, the mRNA expression of *ALB*, *HNF4A*, and *AFP* was 2.3-, 7.1-, and 1.8-fold higher than that in the uninduced SHFSCs group, respectively (Fig. 6c).

## Discussion

HFSCs are adult stem cells with self-renewal ability located in the HF bulge region [2]. Previously, HFSCs have been isolated using tissue adherence or trypsin digestion tissue blocks; however, these methods result in the isolation of non-specific cells with weak activity [38, 39]. Therefore, we selected the dispase II digestion method, which minimize cell damage. Dispase II is a neutral protease used to separate the epidermis from dermis in vitro as it does not damage cell membranes in the tissue [40, 41]. To obtain HFSCs more efficiently, we isolated intact HFs using microdissection from goat skin. Furthermore, HFs could easily be separated from the skin when skin tissues became loosely connected after Dispase II digest. A defining characteristic of HFSCs is adhesion. Particularly, the speed of adhesion is faster than that of other cells to type IV collagen, laminin, and the extracellular matrix [42, 43]. With the differential adhesion of HFSCs, we obtained highly purified HFSCs. This purification method has been verified and applied in previous studies [44, 45]. HFSCs have firmer cell attachment to extracellular matrices and culture plates [46]. Moreover, HFSCs take a longer time to detach from dish surfaces than other cells bytrypisn digestion [47, 48]. We applied different detachment window periods toobtain further purified HFSCs. Therefore, a combination of differential adhesion and detachment was an effective and simple method for purifying PHFSCs and SHFSCs.

HFSC localization and marker expression have been widely studied in humans and mice [21–24]. However, the surface markers of cashmere goat PHFSCs and SHFSCs are relatively unknown. Therefore, we selected conventional surface markers in humans and mice to identify PHFSCs and SHFSCs in Arbas Cashmere goats.

Studies have shown that HF regeneration depends on the activation of *LGR5*-positive HFSCs in the bulge region of HF [24]. Other studies have also shown that *K15* expression can be detected in *LGR5*-positive cells in human and mouse HFs [49]. Similarly, *CD34* are specifically expressed in the HF bulge region [21, 22], which agrees with our current study findings. *K19* has been identified using immunohistochemistry in the mouse HF bulge region, *K19* was confirmed as a marker of HFSCs [20]. Interestingly, *K19* was found to be expression at higher levels in the two types of HFSCs in our study, but little *K19* fluorescent signal could be detected in PDPCs. We speculated that *K19* may not be a specific marker for PHFSCs from cashmere goats. We therefore used multiple surface markers to identify the PHFSCs and SHFSC. In addition, cells expressing *K14* were detected in the bulge region and the basal layer of the epidermis [25]. We provided evidence that HFSCs may be derived from the same source as epidermal cells. In summary, these findings collectively confirmed that the two types of cells isolated and cultured in this experiment were HFSCs.The self-renewal capacity and pluripotency of stem cells are closely related to pluripotent factors, the expression of which determines the function of pluripotent stem cells. *OCT4, SOX2* and *NANOG* are the core factors that regulate cell reprogramming and play a crucial role in stem cell self-renewal and pluripotency [26]. In our study, *OCT4* and *SOX2* were highly expressed in both SHFSCs and PHFSCs. *OCT4* and *SOX2* appear to play a significant role in maintaining the pluripotency of HFSCs. Furthermore, *SOX9*, another transcription factor related to pluripotency, was highly expressed in PHFSCs and SHFSCs. Studies have found that the absence of *SOX9* in HFSCs decreases the expression of pluripotent factors, leading to loss of pluripotency [50].

Previous studies have also discussed the dual potential of HFSCs [17]. To the best of knowledge, most studies on the differentiation of rat and human HFSCs have been performed in bone, neuronal and endothelial tissues [18]. In contrast, few have studies examined the differentiation potential of cashmere goat PHFSCs and SHFSCs [50], the potential of neural differentiation, adipocyte differentiation and hepatic differentiation in two types of HFSCs from cashmere goat is still unknown. Herein, we demonstrated that HFSCs possess multidirectional differentiation potential and can be induced to differentiate into adipocyte-like, neural-like, and liver-like cells.

*NeuN* is a marker of neural differentiation and maturation, which is mainly expressed in the nuclei of mature neurons, and its expression is directly proportional to neuron maturation [28]. *GFAP* is a specific marker of astrocytes in the central nervous system [29]. *MAP2*, a member of the microtubule-associated protein family, acts on microtubule stability and regulates microtubules in axons and dendrites [30]. In previous studies,the neural specific makers NeuN, GFAP and MAP2 expression were always used to evaluate the neural differentiation potential of cells [51, 52]. The results of our neural induction experiments demonstrated that the induced PHFSCs and SHFSCs have morphological changes towards a neural –like appearance, and these markers (*MAP2*, *NeuN* and *GFAP*) were highly expressed in the neural induction group, indicating that the two HFSCs successfully differentiated into nerve cells. The results showed that *MAP2* was expressed before and after induction. Because the cytoskeleton is composed of microtubules and other fibres, normal cultured cells also expressed small amount of *MAP2*.

In general, adipogenic differentiation potential of stem cell was assessed by Oil Red O staining, which specifically stains triglycerides and cholesteryl oleate [53, 54]. In our study, Oil red O staining results showed that lipid accumulation was significantly increased in the induced PHFSCs and SHFSCs. In addition, several adipogenic genes, including ADIPOQ, FABP4, PPARG and Leptin, were used to evaluate the progress of adipocyte differentiation in previous studies [55]. *ADIPOQ* is a bioactive factor that is secreted by the adipose tissue [32]. *FABP4* is primarily expressed in adipocytes, where it maintains lipid homeostasis and plays an important role in regulating fat storage and distribution [56]. *PPARG*, a member of the nuclear hormone superfamily, is a marker of adipogenesis initiation [31]. In our study, the expression of *PPARG*, *ADIPOQ*, and *FABP4* in PHFSCs and SHFSCs increased over time to different degrees. Analysis of adipogenesis-related gene expression showed that both PHFSCs and SHFSCs could differentiate into adipose cells. In addition, *Leptin* accumulation peaked when the two HFSCs were differentiated on day 9. *Leptin* is a protein hormone secreted by the adipose tissue, and its secretion depends on fat accumulation [33]. Therefore, these findings indicate that the accumulation of adipocytes is less before differentiation on the day 9.

Albumin (ALB), Alpha-fetoprotein (AFP) and Hepatocyte nuclear factor 4a (HNF4A) were common hepatocyte-specific genes used to track the differentiation of hepatocyte-like cells [57, 58]. ALB is a marker of mature hepatocytes and is primarily secreted by mature hepatocytes [35]. HNF4A is a highly conserved transcription factor that which regulates hepatocyte phenotype and maintains normal liver function and metabolism [59]. AFP is secreted by the foetal liver, and *AFP* levels decrease during foetal liver development. However, in adults, *AFP* expression may be related to hepatocyte regeneration since *AFP* expression is re-activated through hepatocyte regeneration and hepatocarcinogenesis [34]. Our results showed that *ALB*, *HNF4A* and *AFP* were expressed at high levels in the experimental group. As we all know, an unique function of the liver is to store glycogen [60]. PAS staining revealed that the differentiated PHFSCs and SHFSCs exhibited the similar function of hepatocytes in the present study. The results indicated that the two types of HFSCs could differentiate into hepatocyte-like cells.

Compared with our study, the previous work only isolated and identify goat PHFSCs [61]. However, we not only confirmed specific markers of PHFSCs and SHFSCs in Arbas Cashmere goats, but also successfully first verified that both PHFSCs and SHFSCs possess strong self-renewal capacity and multipotent with the ability to differentiate into adipocyte-like, neural-like, and liver-like cells in specific environments in present work. These characteristics may provide PHFSCs and SHFSCs with unique advantages for stem cell therapy and tissue regeneration.

## Conclusion

In the current study, we successfully isolated and cultured PHFSCs and SHFSCs from Arbas Cashmere goats and identified two types of HFSCs using surface markers. We assessed the expression of pluripotent factors in PHFSCs and SHFSCs and induced HFSCs to differentiate into adipogenic, neurogenic, and hepatic cells. Taken together, our results provide further evidence that the HFSCs of Arbas Cashmere goats are multipotent.

## Methods

### Skin collection and digestion by dispase II

**Fig. 7:**
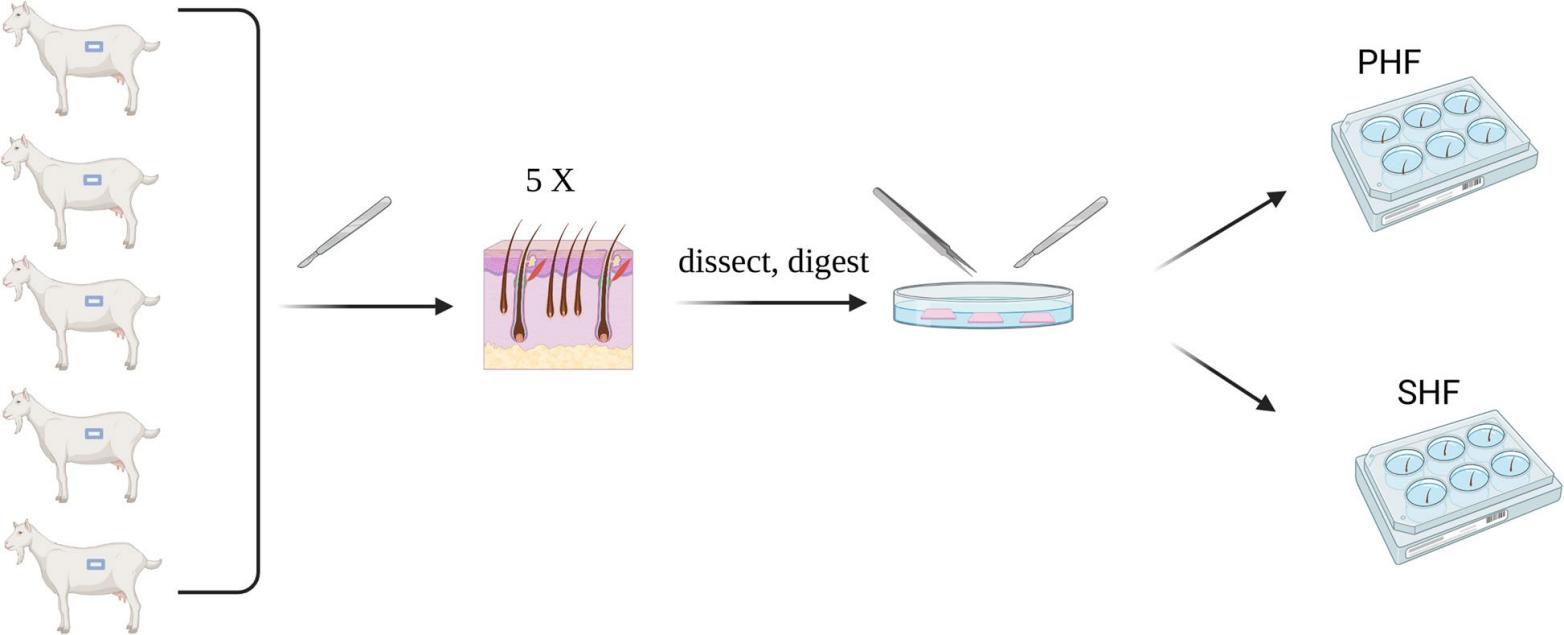
The isolation and culture process of PHFs and SHFs. The schematic shows the isolation and culture process of PHF and SHF from Arbas Cashmere goats

A small area on the animals’ flank skin was excised from five 2-year-old female Arbas Cashmere goats. Five samples were then shaved with a stripping knife, washed, sterilised with alcohol, and washed with Dulbecco’s phosphate-buffered saline (DPBS) (BI, Israel) containing 1% penicillin–streptomycin solution (03–031-1BCS, BI, Israel). These skins samples were then randomly dissected with a scalpel into 3mm^*2*^ strips along the direction of the hair fibre (Fig. 7). After 90 min of digestion in 0.25% dispase II (D4694, Sigma, USA) at 37℃, the skin tissues were washed with DPBS.

### Skin microdissection and HFSCs isolation

Primary hair follicles (PHFs) and second hair follicles (SHFs) were microdissected from the skin using tweezers and scalpel as previously described [62, 63]. These skin strips were placed in a dish (430,167, Corning, USA) containing DPBS with 1% penicillin–streptomycin solution. The skin was then held with tweezers, and the PHF and SHF were removed from the tissue using scalpel under a stereomicroscope (SMZ800N, NIKON, Japan) (Fig. 7). The isolated PHFs and SHFss were placed separately in 6-well culture plates (3516, Corning, USA) and cultured in HFSC medium, comprising Dulbecco’s modified Eagle medium (DMEM)/F12 (1:1) medium (BI, Israel) with 4% (v/v) foetal bovine serum(FBS) (BI, Israel), 1% penicillin–streptomycin mixture, 14 ng/mL epidermal growth factor (EGF) (315–9-100, Pepro Tech, USA), 0.4 ng/mL hydrocortisone (H0888, Sigma, USA), and 0.5 µg/mL Insulin-Transferrin-Selenium (ITS) (51,500–56, GIBCO, USA).. Approximately 5–10 HFs were placed in each well of 6-well plates. Medium was changed every 2 days. The PHFSCs and SHFSCs were separately migrated from the PHFs and SHFs on day 1. The PHFSCs and SHFSCs were collected by trypsinization, after 4 days of culture.

### HFSCs purification

The primary PHFSC and SHFSC purification methods were similar. Primary HFSCs were purified according to a previously described protocol [45] with slight modifications. Briefly, collagen type IV (C5533, Sigma, USA) was dissolved at a concentration of 0.1 mg/mL according to the manufacturer’s instructions. Subsequently, 6-well culture plates were coated with Collagen IV at 37 °C in an atmosphere of 5% CO_2_ (v/v) for approximately 1 h. Then the collagen solution was then removed and the plates were washed three times with DPBS.

The PHFSCs and SHFSCs collected from previous step were separately resuspended in a culture medium and seeded on collagen-coated plates. After 20 min, non-adherent cells were discarded, and fresh culture medium was added to culture adherent cells.

The secondary purification procedure was performed via different detachment [64]. Briefly, adherent cells in the plates with 90% confluency were trypsinized by 0.25% trypsin for 2 min at 37 °C, and partial spindle cells were discarded, which became round and began to easily detach from the plates surface. The remaining cobblestone-like cells, which were tightly attached to the plates, were harvested using an additional 0.25% trypsin for 5 min. The collected cells continue to be resuspended and planted on the collagen-coated plate. These methods were repeated twice to obtain purified HFSCs.

### HFSCs culture and passaging

Two types of purification HFSCs were collected and resuspended in HFSC culture medium, then placed into 6-well culture plates. PHFSCs and SHFSCs were cultured for 3–5 days at 37 °C in an atmosphere of 5% CO_2_ (v/v). The medium was changed every 3 days until PHFSCs and SHFSCs reached approximately 80% confluency. Purified PHFSCs and SHFSCs were digested again using trypsin for passaging.

### Giemsa staining

After purification, PHFSCs and SHFSCs were passaged five times. PHFSCs and SHFSCs were washed two to three times with DPBS and then fixed with methanol for 2–3 min. Giemsa concentrate (1 mL;Solarbio, China) and diluent (9 mL;Solarbio, China) were thoroughly mixed. PHFSCs and SHFSCs were then incubated in a 6-well culture plate with the diluted staining solution for 30 min, after which they were washed slowly with ultrapure water and examined under a microscope (NIKON ECLIPSE Ti2).

### Cell viability and proliferation assessment

Cell Counting Kit-8 (CCK-8, Yesen, China) was used to evaluate cell viability and proliferation, following the manufacturer’s instructions. PHFSCs and SHFSCs (2 × 10^3^ cells/well) were seeded separately in 96-well plates for 12 h and 10μL of CCK-8 solution was added to each well and incubated for 2 h at 37 °C in a 5% CO^2^ (v/v) atmosphere. The absorbance was measured to evaluate cell viability using a microplate reader (Varioskan Flash, Thermo Fisher Scientific, USA). And the same method was performed continunously for 7 days to evaluate cell proliferation assessment.

### Immunofluorescence for PHFSCs, SHFSCs, PDPCs and SDPCs characterization

Fifth passage PHFSCs, SHFSCs, PDPCs and SDPCs (1 × 10^5^ cells/well) were seeded on glass coverslips placed in a 24-well culture plate (3527, Coning, USA) and fixed with 4% paraformaldehyde. Next, cells were permeabilised for 15 min using 0.5% Triton X-100. After washing the cells with DPBS, non-specific antigen-binding was blocked by incubating cells with a blocking buffer containing 5% bovine serum albumin (BSA) (SW3015, Solarbio, China) for 1 h. Cells were then incubated overnight with the primary antibodies at 4 °C. Cells were then washed three times with phosphate-buffered saline containing Tween 20(PBST) (P1031, Solarbio, China) for 10 min each. Cells were incubated with the secondary antibody (Donkey Anti-Rabbit IgG H&L (Alexa Fluor® 488), diluted 1:1000, Abcam, England) for 60 min, and then mounted using a mounting medium containing DAPI (Solarbio, China). Cells were placed in the dark prior to observation under a fluorescence microscope (Nikon-Air, Nikon Iinstruments Co., LTD, Japan). Primary dermal papilla cells (PDPCs) and secondary dermal papilla cells (SDPCs) were isolated, identified and preserved in the laboratory [42]. Fifth passage PDPCs and SDPCs were used as a control for the PHFSCs group and SHFSCs group, respectively. The primary antibodies used in this step and their dilution were as follows: Rabbit Anti-CD34 antibody (Bioss, China) [1:300], Rabbit Anti-K14 antibody (Bioss, China) [1:300], Rabbit Anti-K15 antibody (Bioss, China) [1:200], Rabbit Anti-K19 antibody (Bioss, China)[1:300], Rabbit Anti-LGR5 antibody (Bioss, China) [1:300], Rabbit Anti-SOX9 antibody (Bioss, China) [1:300], Rabbit Anti-OCT4 Polyclonal Antibody (Proteintech, USA) [1:300], Rabbit Anti-SOX2 Polyclonal Antibody (Proteintech, USA) [1:200].

### Reverse transcription and quantitative real-time PCR (qRT-PCR)

Fifth passage PHFSCs, SHFSCs, PDPCs and SDPCs were harvested using trypsinisation and centrifugation, and cell pellets were washed twice with ice-cold DPBS. Total RNA was obtained from HFSCs using the RNAiso Plus (Takara, Japan) following the manufacturer’s instructions. The cell pellets were lysed with 1 mL RNAiso Plus solution at 25 °C. Chloroform was then added to the mixture of cell pellets and RNAiso Plus solution after 5 min. The mixture was then vortexed and centrifuged again at 4 °C for 15 min (12,000 rpm). Next we collected the supernatant added isopropanol, and mixed the solution thoroughly. The supernatant was removed after centrifuging. Finally, 70% ethanol was added to wash the sample once and was discarded after centrifuging. The sediment was resuspended in RNase-free water.

The quantity and quality of the total RNA were measured using NanoDrop™ One (Thermo Fisher Scientific, USA). RNA purity of the samples was checked using a OD260/280 ration by NanoDrop with an adequate range from 1.8 to 2.1. The concentrations of RNA extracted were approximately 600-1000 ng/μL, and the amount of extracted RNA extracted was ~ 12,000–20,000 ng.

mRNA was reverse transcribed using PrimeScript RT-PCR kit with gDNA Eraser (Takara, Japan) following the instructions. After reverse transcription, the cDNA obtained was treated with RNase H (TransGen Biotech, China). The quantity and quality of cDNA were assessed using a NanoDropTM One. The quantity of cDNA was approximately 1,000 ng, and the quality of cDNA was determined with the OD260/280 ratio, which was in the range of 1.8–2.0. The primers used for qRT-PCR are listed in Table 1. qRT-PCR was performed using TB Green Premix Ex Taq II (TilRNaseH Plus,Takara, Japan). According to the manufactures instructions, each reaction volume was 20μL, including 10μL of SYBR regent, 0.4μL of each primer (10 μm), 7.2μL of RNase-free water, and 2μL of cDNA template. The quantity of cDNA template that was added was < 100 ng. Target gene expression was evaluated in triplicate using the 2^–ΔΔCt^ method [65] and was normalised to GAPDH levels. Fifth passage PDPCs and SDPCs were used as a control for the PHFSC group and SHFSC group, respectively.

**Table 1:** The primers of HFSCs markers

Gene name	Forward primers(5’-3’)	Revers primers(5’-3’)
GAPDH	GGTCGGAGTGAACGGAT	TCTGCCTTGACTGTGCC
CD34	CCAGCCCTGTGACTTCTTCCA	CAGACTCGGGTCAGCTTCTC
K14	GCTATGGCGGTGGTTTCA	CCAGGGCACGCACTTTGT
K15	CGTGCTGTCAGAAATGAGGGAG	GCTTTCATGCTGAGCTGGGACT
K19	TGCTCCGGGCATCGACCTA	GCCAGCGACCTCCTTGTTC
LGR5	AATTCGCTTTGCTTCCTCGT	ATGCTCTCCAGGTCTCCCTTT
SOX9	AGTACCCGCACCTGCACAA	CCGTTCTTCACCGACTTCCTC
OCt4	GCCAAGCTCCTAAAGCAGAAGA	AAAGCCTCAAAACGGCAGATAG
SOX2	CGCCGAGTGGAAACTTTTGT	CTCCAGGCAGTGTGTACTTATCCTT

### Western blotting

Total protein was extracted from fifth passage PHFSCs, SHFSCs, PDPCs and SDPCs using the Mammalian Protein Extraction Kit (CWBIO, China), following the manufacturer’s instructions. Protein concentration was evaluated using the BCA Protein Assay Kit (Solarbio, China). Total protein was denatured by boiling in SDS Loading Buffer (CWBIO, China) for 5–10 min. Equal amounts of protein were loaded onto SDS-PAGE gels and resolved using electrophoresis, and then the gel was cut out a strips according to size of target proteins. Subsequently, they were transferred onto a nitrocellulose membrane (Millipore). The membrane was cut into the same size as the gel strip and blocked with 5% skimmed milk for 1 h, and then incubated overnight with primary antibodies in a shaker at 4 °C. After 12 h, the membrane was washed three times with TBST and incubated with HRP-conjugated secondary antibody (Proteintech, USA, diluted 1:10,000) for 1 h at 25 °C. Bands were visualised using ECL Western Blotting Substrate (Thermo Fisher Scientific, USA). Band intensity was normalised to that of *GAPDH*. All primary antibodies used in this step and their dilution were as follows: Rabbit Anti-CD34 antibody (Bioss, China) [1:500], Rabbit Anti-CK14 antibody (Bioss, China) [1:1,000], Rabbit Anti-LGR5 antibody (Bioss, China) [1:1,000], Rabbit Anti-K15 antibody (Bioss, China) [1:500], Rabbit Anti-K19 antibody (Bioss, China)[1:1,000], Rabbit Anti-SOX9 antibody (Bioss, China) [1:500], OCT4 Polyclonal Antibody (Proteintech, USA) [1:500], SOX2 Polyclonal Antibody (Proteintech, USA), [1:500], GAPDH Polyclonal Antibody (Proteintech, USA) [1:10000]. Fifth passage PDPCs and SDPCs were used as controls for the SHFSCs and PHFSCs groups, respectively.

### Adipogenic differentiation

Fifth passage PHFSCs and SHFSCs were cultured separately in 6-well plates at a density of 1 × 10^5^ cells per well until reaching 60–70% confluency, as described by Wang et al. [66].They were then incubated in an adipocyte pre-induction medium (4% FBS, 5% rabbit serum, 33 µM biotin, 5 µM rosiglitazone, 1 µM dexamethasone, 17 µM pantothenic acid, 0.5 mM 3-isobutyl-1-methylxanthine, and 1 µM insulin) for 3 days. The pre-induction medium was then replaced with a maintenance differentiation medium that did not contain 3-isobutyl-1-methylxanthine or rosiglitazone. The differentiated PHFSCs and SHFSCs were used as the experimental groups (induced groups). Fifth passage PHFSCs and SHFSCs were cultured under normal culture conditions and were used as the control group (uninduced group). Then induced and uninduced groups were fixed at three-time points (days 6, 9 and 12) with 4% paraformaldehyde for 10 min, and then stained with fresh 0.5% Oil Red O for 20 min. The formation of lipid droplets was observed under a microscope.

Total RNA was extracted from differentiated PHFSCs and SHFSCs collected on days 6, 9, and 12. *ADIPOQ*, *FABP4*, *PPARG* and *Leptin* mRNA expression was detected in the samples of induced and uninduced groups. The primer sequences of all above genes are shown in Table 2.

**Table 2:** The primer sequences of Adipogenic differentiation maker

Gene name	Forward primers(5’-3’)	Revers primers(5’-3’)
ADIPOQ	CAGGTTGGATGGCAGGCATT	CCTTAGGACCAACAAGACCTGG
FABP4	TGACAGGAAAGTCAAGAGCATC	CTCTGGTGGTAGTGACACCG
PPARG	TCTTCCGGAGGACGATCAGA	CCCGAACCTGATGGCGTTAT
Leptin	GACATCTCACACACGCAGTCC	CTGGCGAGGATCTGTTGGTAG

### Neurogenic cell differentiation

Fifth passage PHFSCs and SHFSCs were individually cultured in 6-well plates at a density of 1 × 10^5^ cells per well until reaching 60–70% confluency. Two types of HFSCs were then incubated with nerve pre-induction medium (5% FBS and 1 mM β-mercaptoethanol) for 1 day, which was subsequently, replaced with a nerve differentiation medium containing 11 µM β-mercaptoethanol (Solarbio, China). The PHFSCs and SHFSCs were then incubated for 28 h and observed under a microscope. Differentiated PHFSCs and SHFSCs were used as the experimental groups (induced groups). Fifth PHFSCs and SHFSCs were cultured under a normal culture condition and were used as the control group (uninduced group).

Immunofluorescence was performed to evaluate the expression of *NeuN*, *MAP2*, and *GFAP* in induced and uninduced groups. The method of immunofluorescence was described in Sect. 4.4.

We collected differentiated PHFSCs and SHFSCs at 28 h and extracted total RNA. *NeuN*, *GFAP* and *MAP2* mRNA expression were detected in the samples of induced and uninduced groups. The primer sequences of the genes are shown in Table 3. All primary antibodies used in this step and their dilution factors were as follows: NeuN Polyclonal Antibody (Protein tech, USA) [1:300], GFAP Polyclonal antibody (Protein tech, USA) [1:300], Rabbit Anti-MAP2 antibody (Bioss, China) [1:300].

**Table 3:** The primer sequences of Neurogenic cell differentiation maker

Gene name	Forward primers(5’-3’)	Revers primers(5’-3’)
MAP2	CGTTTCCGCGCCCAGATT	GATGTTTCCTTTTAAT
GFAP	GGGGAAAGTCACAAGGTCACA	TGTCCAGGCTGGTTTCGAG
NeuN	CTCAGAACCACCTCACCCAC	ACTGTGGGCTCTCTGTTTGC

### Liver-like cell differentiation

Fifth passage PHFSCs and SHFSCs were cultured separately in 6-well plates at a density of 1 × 10^5^ cells per well until reaching 60–70% confluency, as described by Shi et al. [67]. Two types of HFSCs were then incubated with liver pre-induction medium (10 ng/mL basic fibroblast growth factor (bFGF) and 20 ng/mL EGF) for 2 days. Next, the medium was replaced with a liver differentiation medium containing 10 ng/mL bFGF (Pepro Tech, USA), 20 ng/mL hepatocyte growth factor (Pepro Tech, USA), and 0.61 g/L nicotinamide for 3 days. After 3 days, the differentiation medium was replaced with a liver cell maturation medium containing 1 µM dexamethasone and 20 ng/mL Oncostatin M (OSM) (Pepro Tech, USA) as well as 50 ng/mL ITS for 14 days. The differentiated PHFSCs and SHFSCs were used as the experimental groups (induced groups). Fifth passage PHFSCs and SHFSCs were cultured under a normal culture condition and used as the control group (uninduced group).

Glycogen Periodic Acid Schiff (PAS) (Solarbio, China) staining was performed according to the manufacturer’s instructions. Differentiated PHFSCs and SHFSCs were collected on day 14, from which total RNA was extracted. *ALB*, *AFP* and *HNF4*A mRNA expression was detected in the samples of induced and uninduced groups. The primer sequences for genes are shown in Table 4.

**Table 4:** The primer sequences of Liver-like cell differentiation maker

Gene name	Forward primers(5’-3’)	Revers primers(5’-3’)
AFP	CTGTCCTGTATGCACCTA	CAGTTATGGCTCGGAAG
ALB	GATGAGCCTCAGAATTTAA	GGTGCTTTCCTGGTGTA
HNF4A	GTACGCCTGCCTCAAAGCC	AGTCATACTGGCGGTCGTTG

### Statistical analysis

All experiments were performed in triplicate. The grey values corresponding to the bands on the western blots were calculated using ImageJ (Image J1.5 software, NIH, USA). All graphs and data were prepared and analysed using GraphPad Prism 8 (GraphPad 8.0.1 software, San Diego, USA). All data were subjected to a normality test (Shapiro–Wilk tests) and presented as mean ± standard deviation. Differences between the groups were analysed using a Student’s t**-**test, and the data homoscedasticity was assessed using the variance F**-**test. *P***-**values < 0.05 were considered statistically significant.

## Supplementary Information

**Additional file 1: Supplementary Fig. 1.** Original western blotting of Fig2. and Fig3. The full**-**length blots show specific protein bands marked in the figure. **Supplementary Fig. 2.** Original western blotting of Fig. 4. The full-length blots show specific protein bands marked in the figure. Line1: induced group; Line2: uninduced group; Line 3: induced group; Line 4: uninduced group. All Western Blot experiments were repeated three times.

## Data Availability

All data generated or analysed during this study are included in this published article [and its supplementary information files].

## Abbreviations

HFSCs: Hair follicles stem cells
PHFSCs: Primary hair follicles stem cells
SHFSCs: Secondary hair follicles stem cells
HF: Hair follicle
PHFs: Primary hair follicles
SHFs: Secondary hair follicles
DPCs: Dermal papilla cells
PDPCs: Primary dermal papilla cells
SDPCs: Secondary dermal papilla cells
ALB: Albumin
HNF4A: Hepatocyte nuclear factor 4a
AFP: Alpha**-**fetoprotein
DPBS: Dulbecco’s phosphate**-**buffered saline
DMEM: Dulbecco’s modified Eagle medium
FBS: Foetal bovine serum
EGF: Epidermal growth factor
ITS: Insulin**-**Transferrin**-**Selenium
bFGF: Basic fibroblast growth factor
OSM: Oncostatin M
PAS: Glycogen Periodic Acid Schiff
