# Supplementary material for: Neural, adipocyte and hepatic differentiation potential of primary and secondary hair follicle stem cells isolated from Arbas Cashmere goats
Source: BMC Vet Res. 2022 Aug 15;18:313. doi: 10.1186/s12917-022-03420-3 (PMC9377108; doi:10.1186/s12917-022-03420-3)

Supplementary Fig. 1: Original western blotting of Fig2. and Fig3. The full-length blots show specific protein bands marked in the figure.

Reapeate1

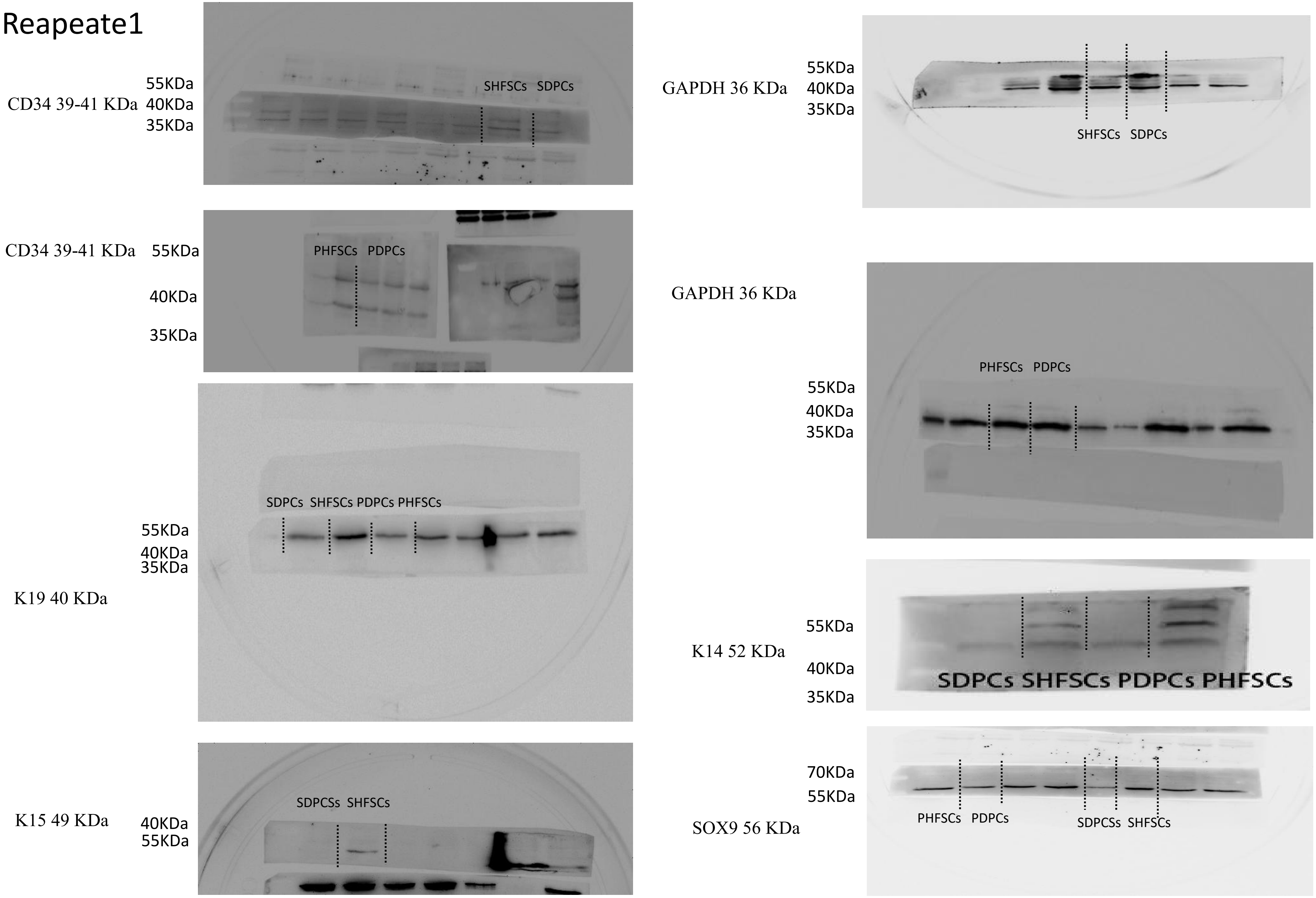

K15 49 KDa

55KDa
40KDa

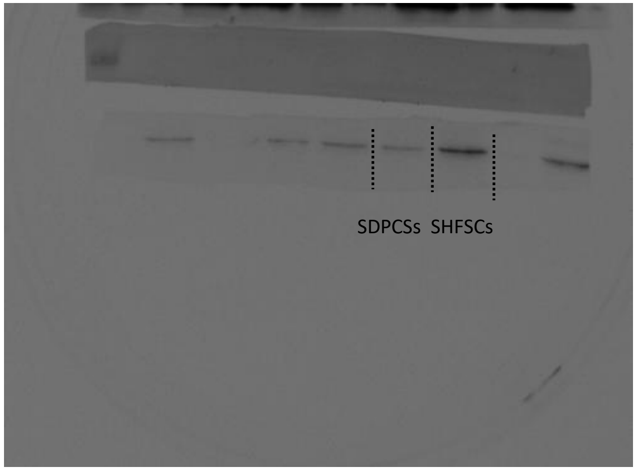

LGR5 98 KDa

100KDa
70KDa

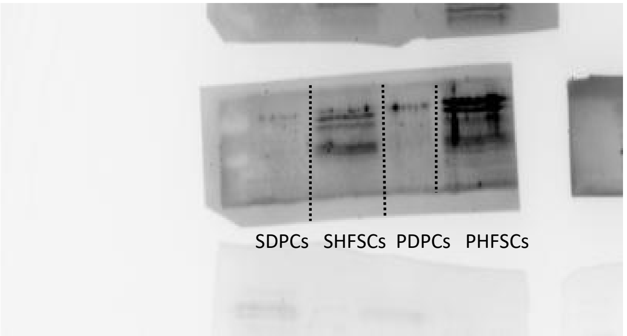

OCT4 39 KDa

40KDa
35KDa

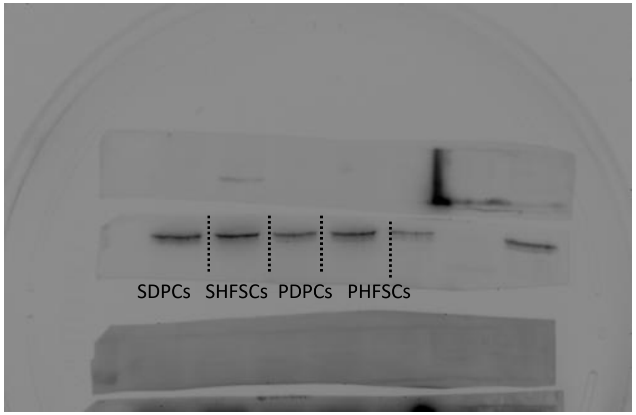

SOX2 34 KDa

35KDa
25KDa

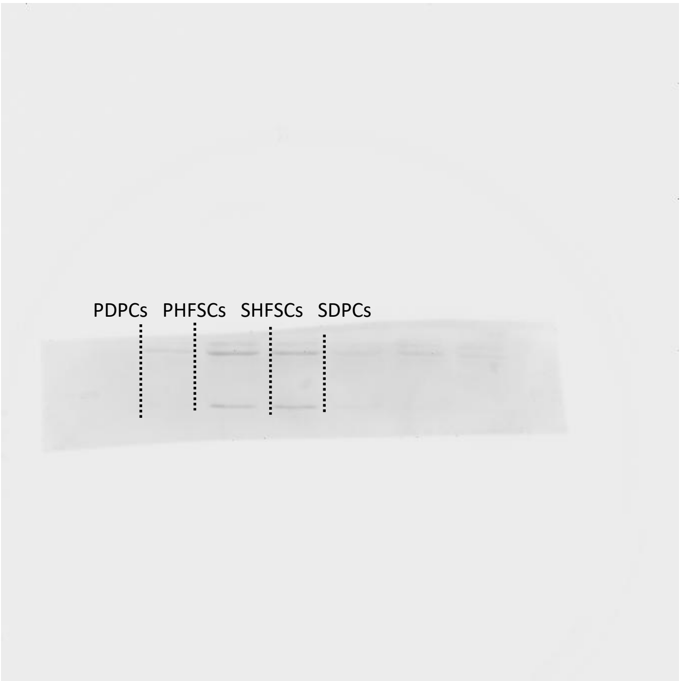

Reapeate2

CD34 39-41 KDa

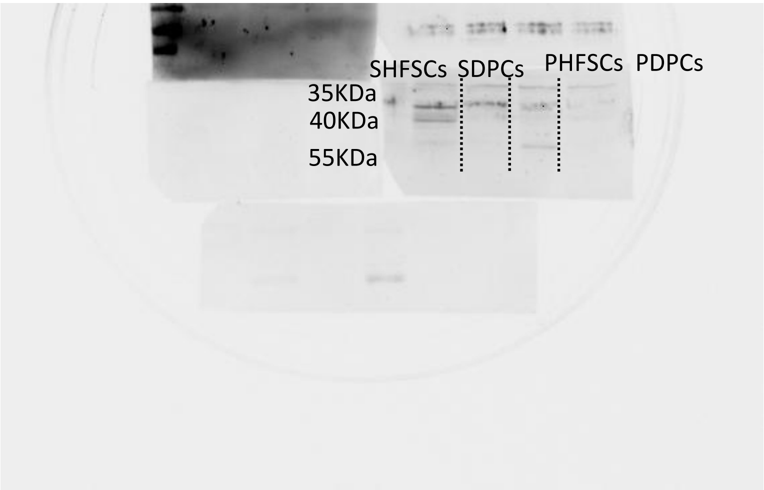

GAPDH 36 KDa

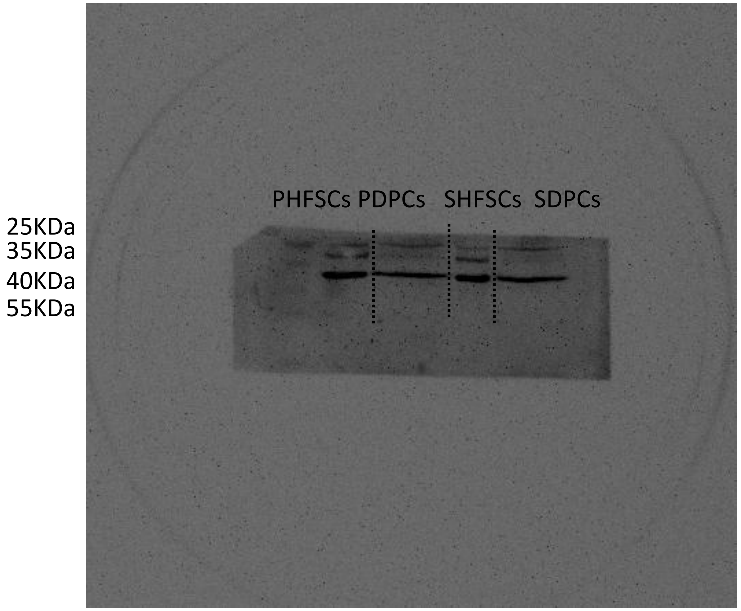

K19 40 KDa

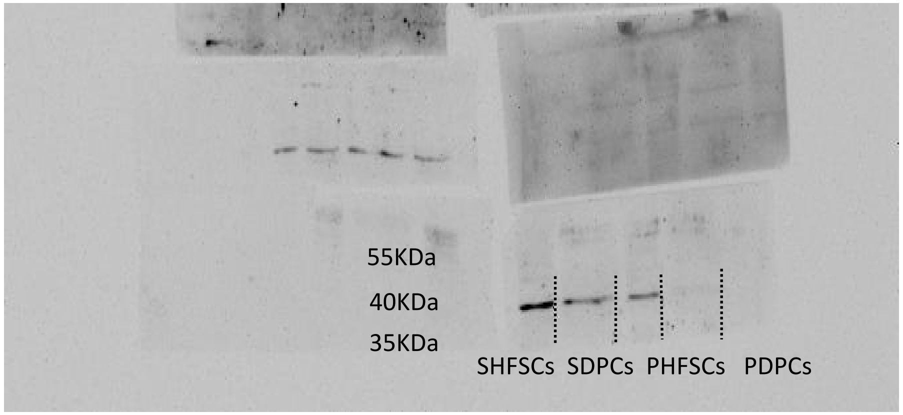

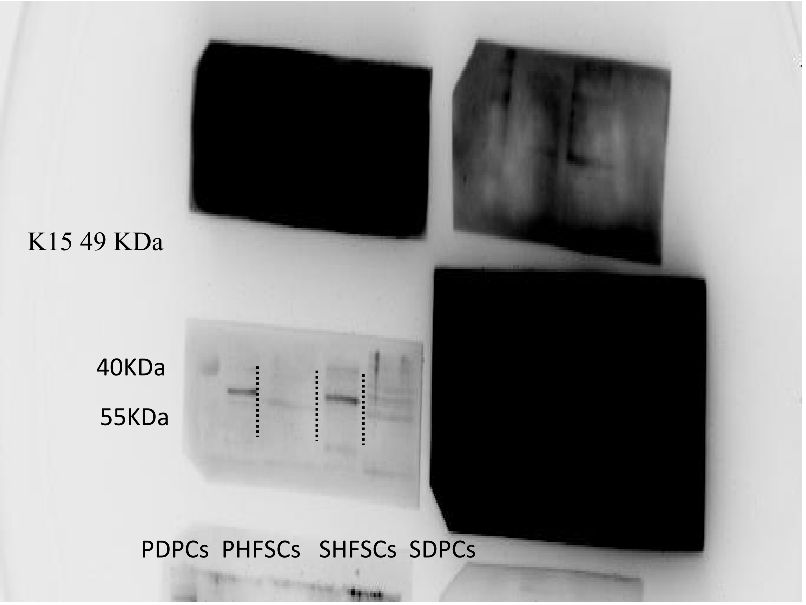

LGR5 98 KDa

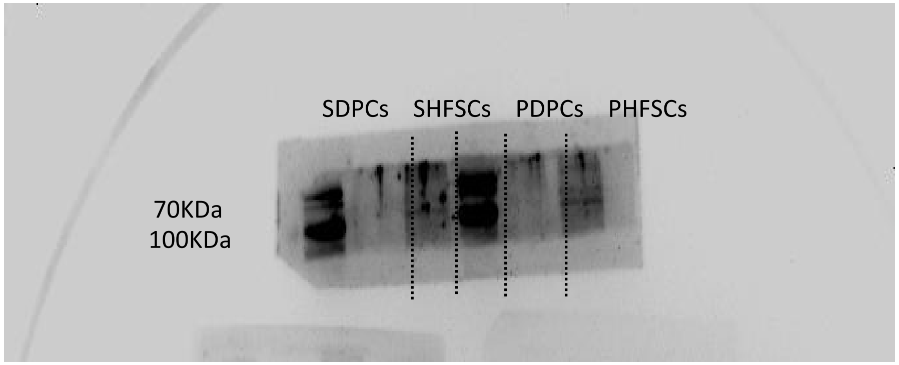

K14 52 KDa

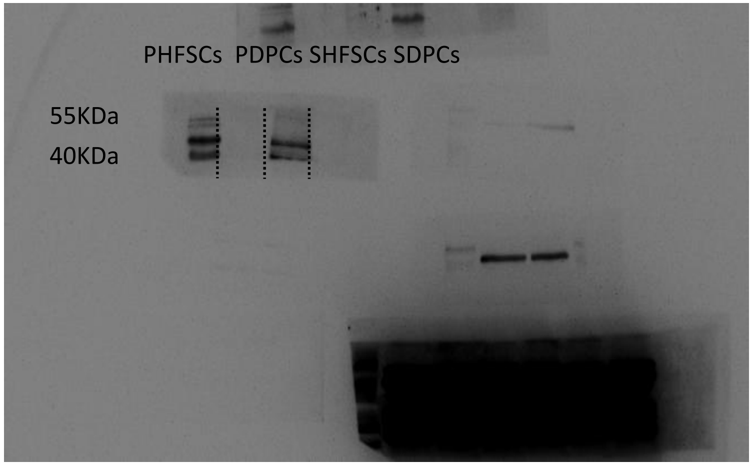

SOX9 56 KDa

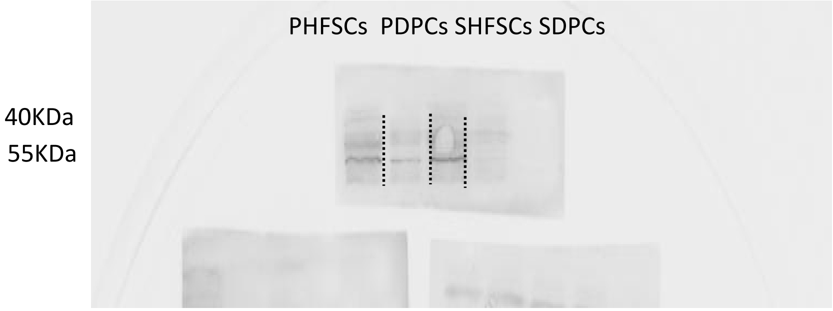

SOX2 34 KDa

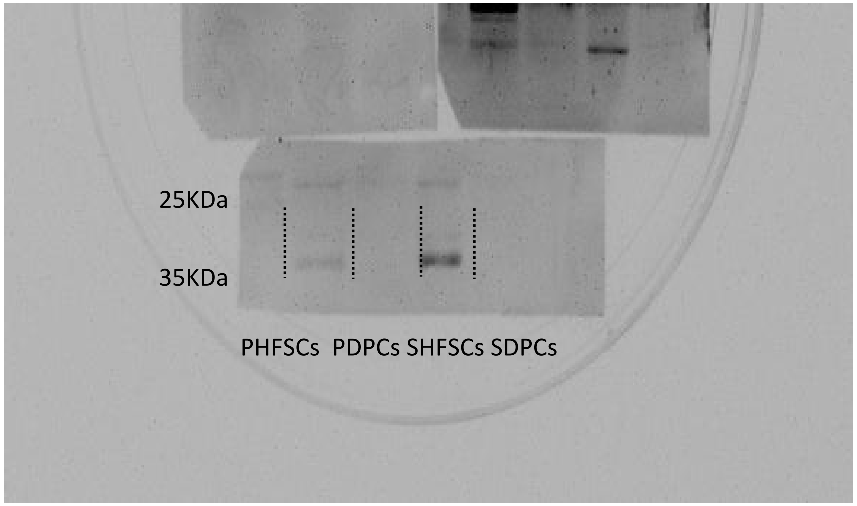

OCT4 39 KDa

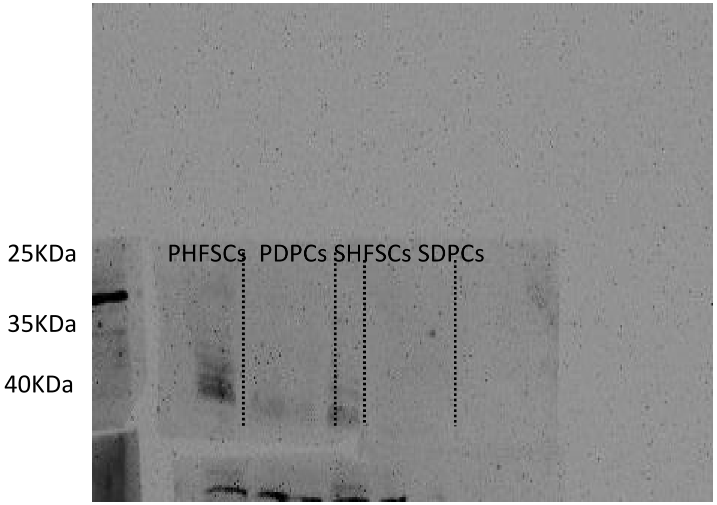

Reapeate3

CD34 39 KDa

40KDa
35KDa

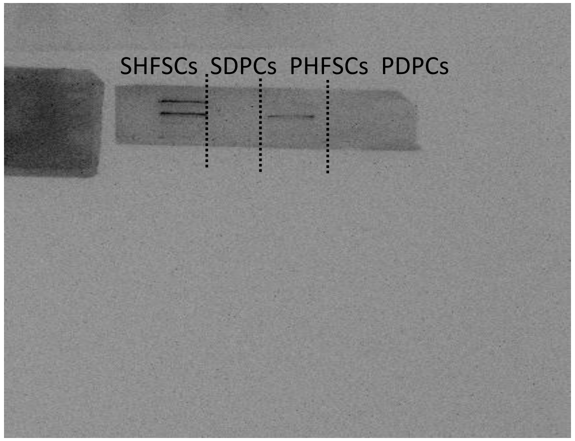

GAPDH 36 KDa

35KDa
40KDa
55KDa

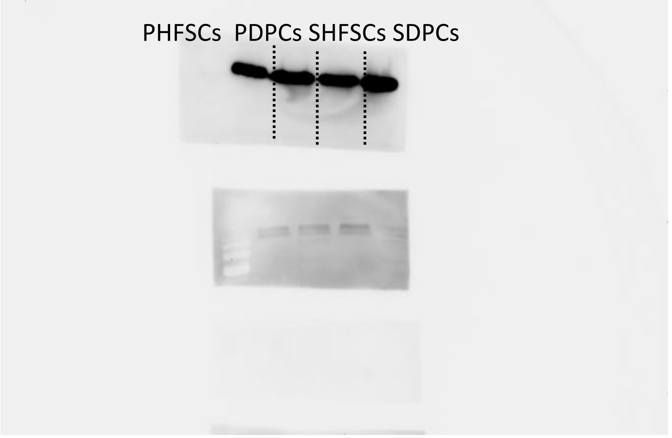

K19 40 KDa

35KDa
40KDa

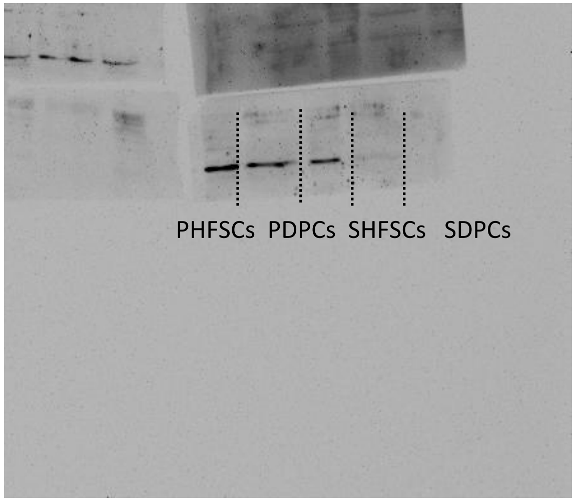

LGR5 98 KDa

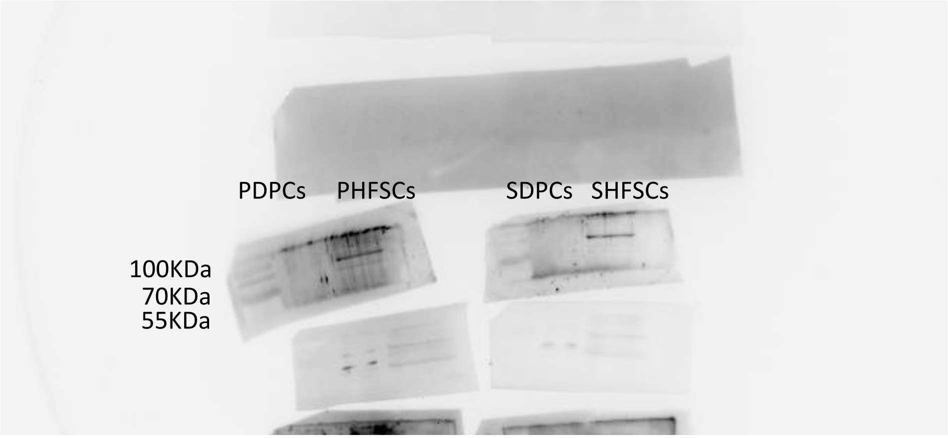

K15 49 KDa

40KDa
55KDa

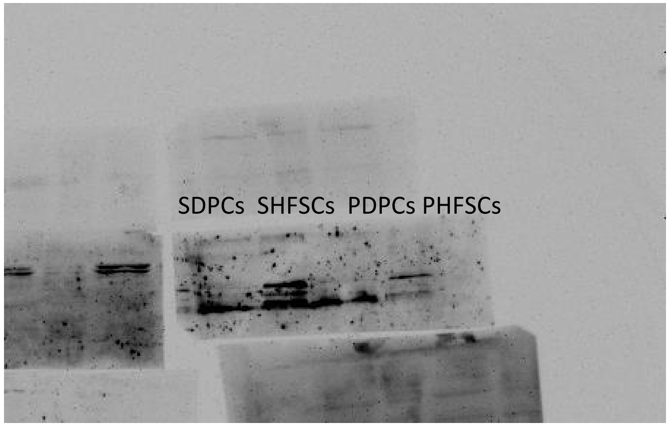

K14 52 KDa

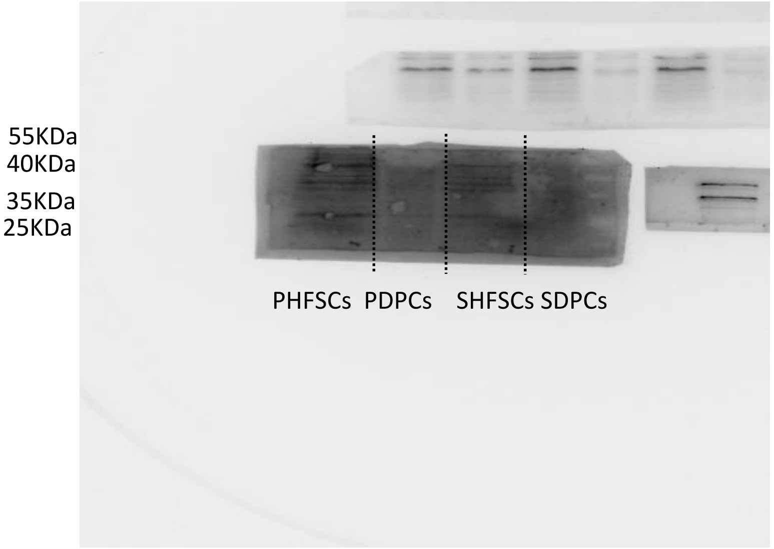

SOX2 34 KDa

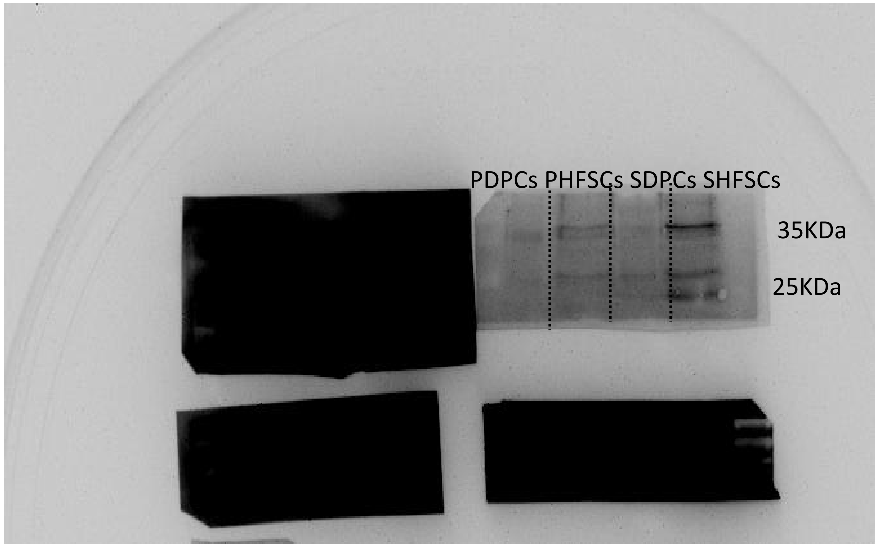

SOX9 56 KDa

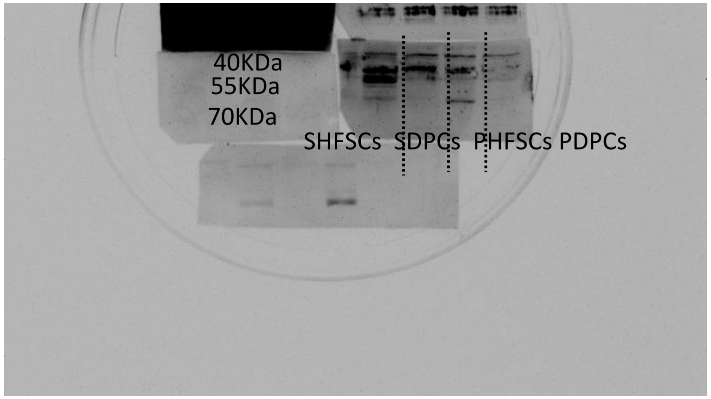

OCT4 39 KDa

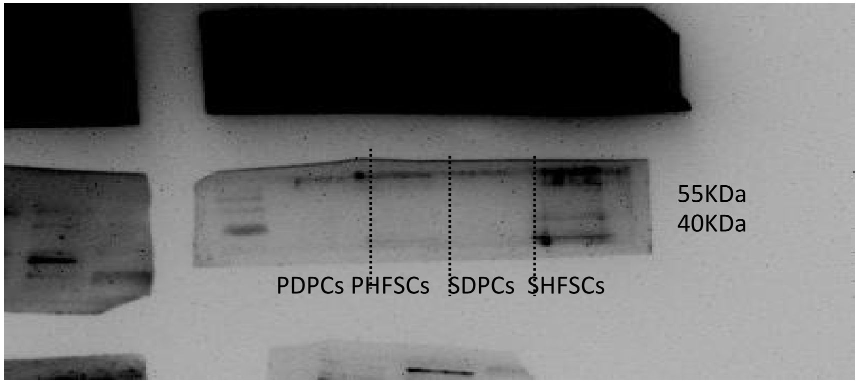

Supplementary Fig. 2: Original western blotting of Fig. 4. The full-length blots show specific protein bands marked in the figure. Line1: induced group; Line2: uninduced group; Line 3:induced group; Line 4: uninduced group. All Western Blot experiments were repeated three times.

Reapeate1

GAPDH 36 KDa

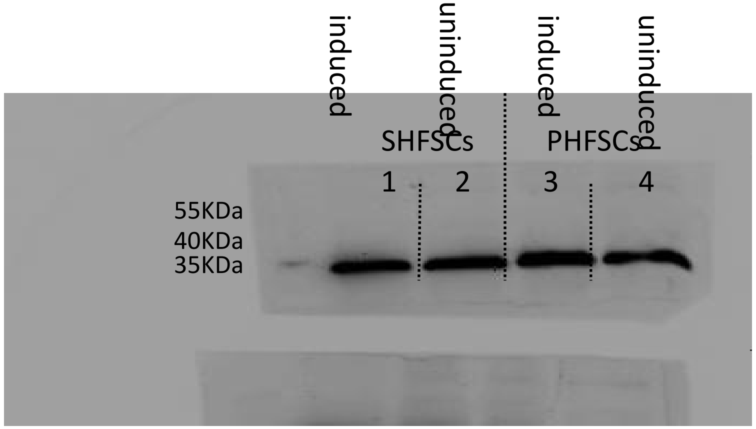

MAP2 72 KDa

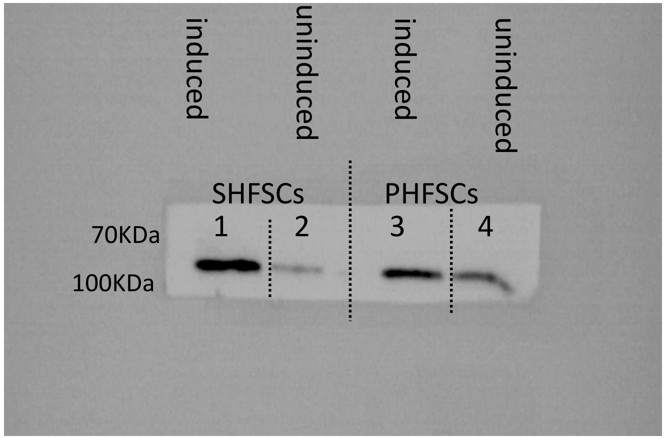

NeuN 46-52KDa

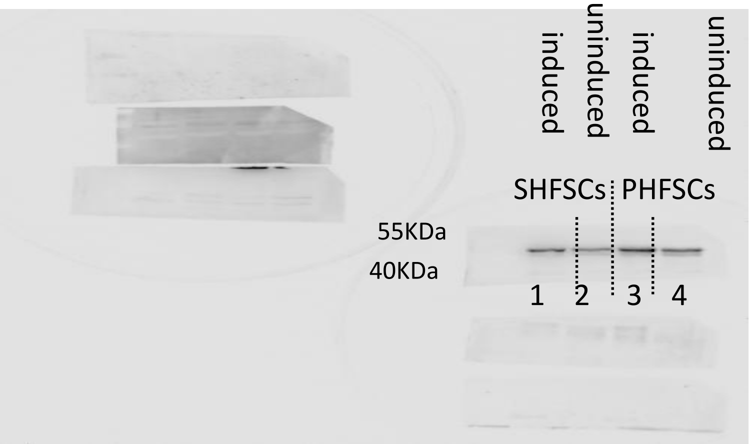

GFAP 45-50KDa

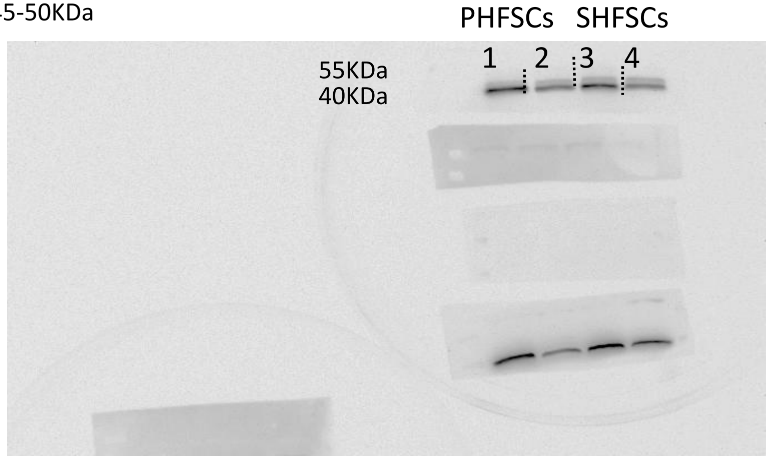

Reapeate2

GAPDH 37 KDa

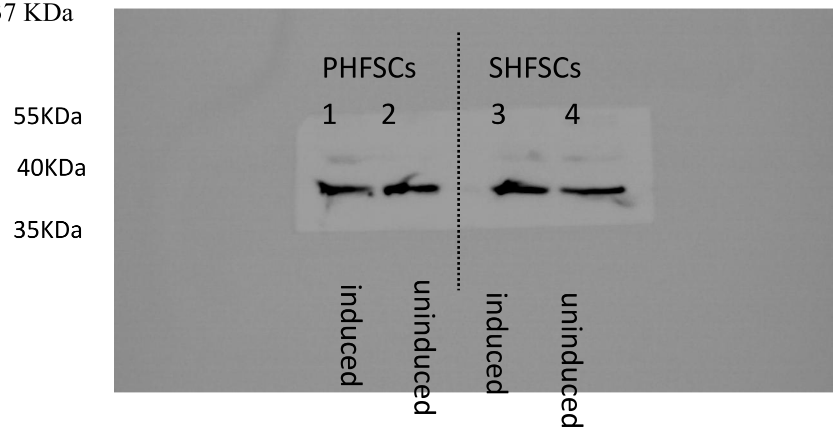

NeuN 46-52KDa

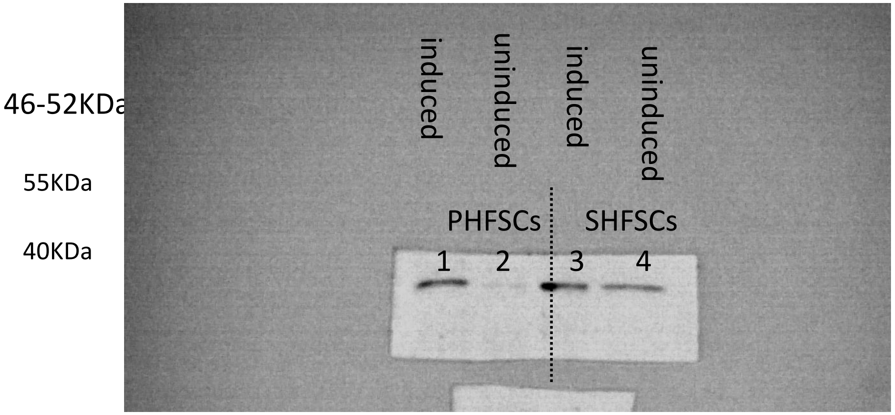

GFAP 45-50KDa

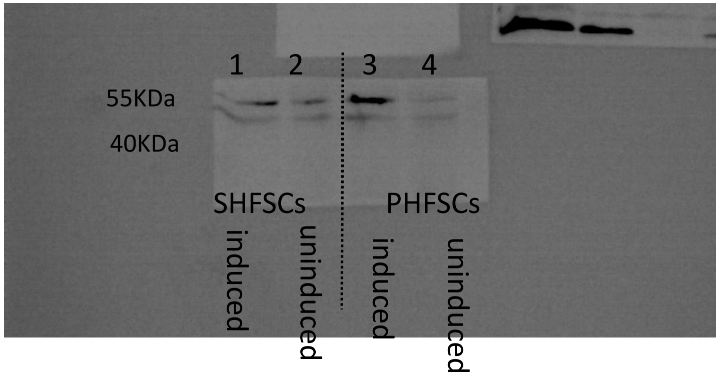

MAP2 72 KDa

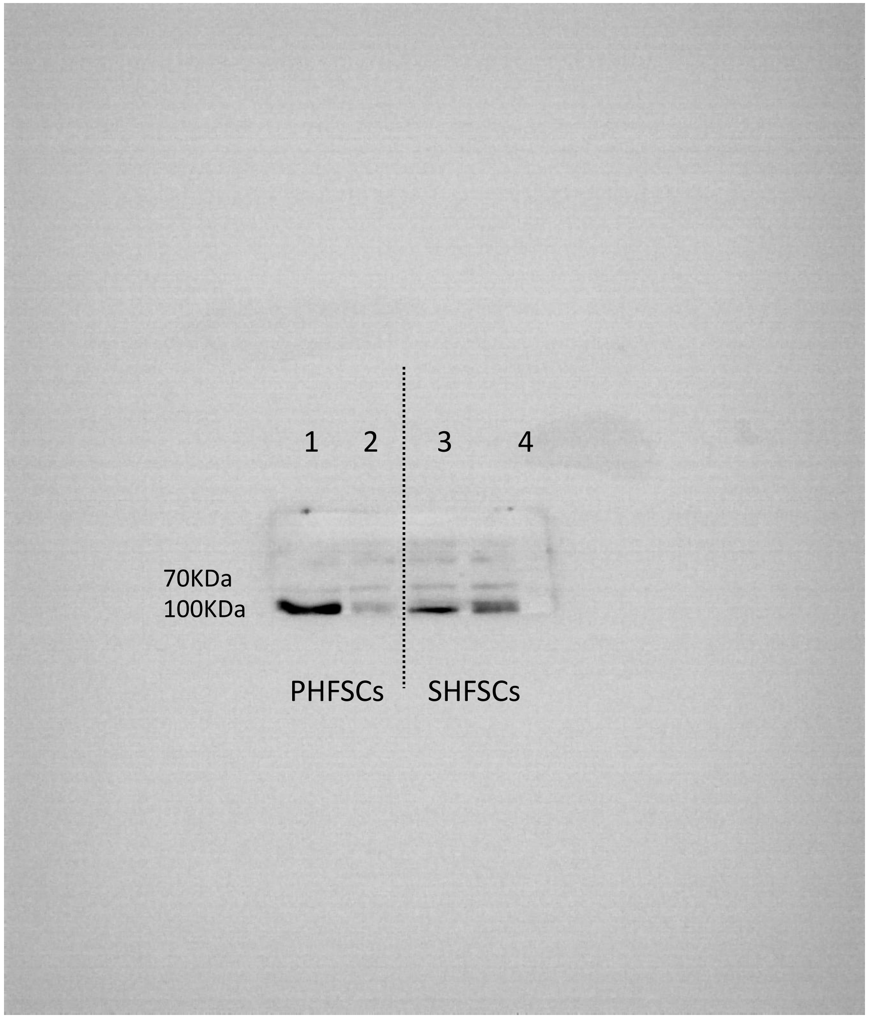

Reapeate3

GAPDH 37 KDa

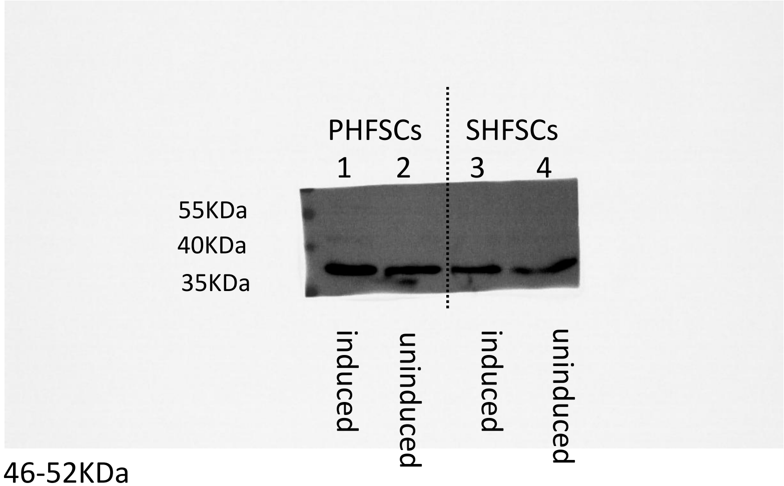

GFAP 45-50KDa

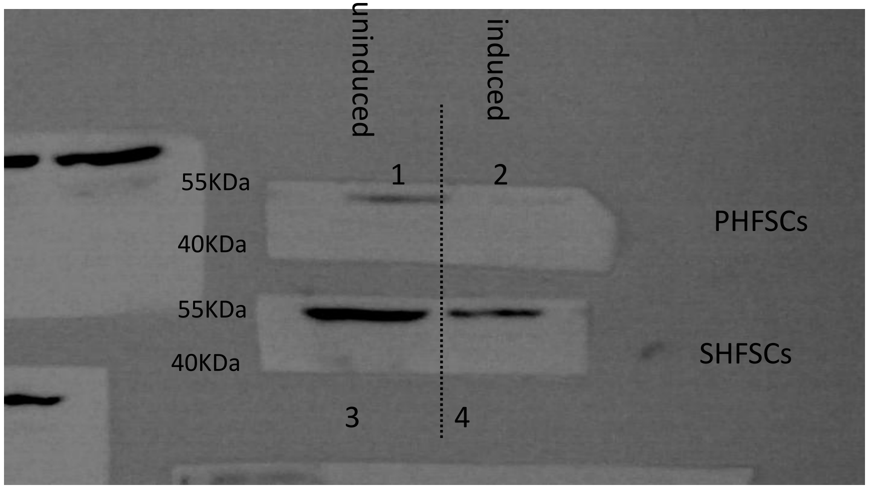

NeuN 46-52KDa

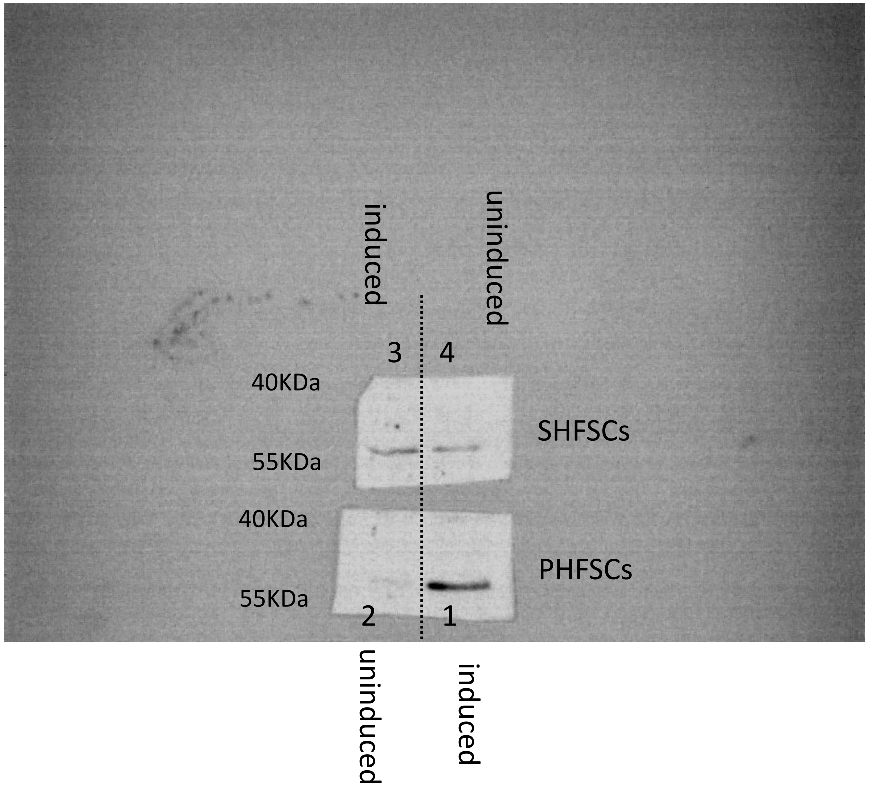

MAP2 72 KDa

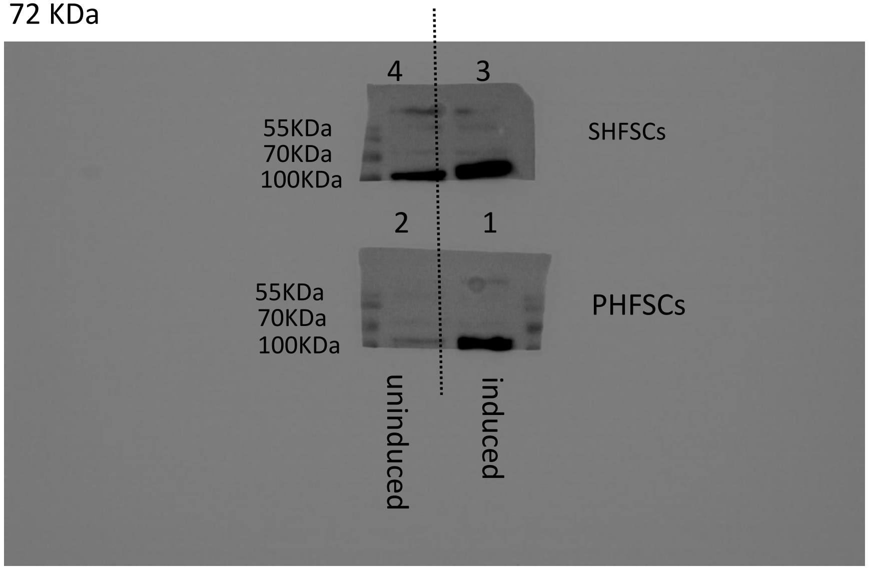

Supplement: Supplementary file 1 — Additional file 1: Supplementary Fig. 1. Original western blotting of Fig2. and Fig3. The full-length blots show specific protein bands marked in the figure. Supplementary Fig. 2. Original western blotting of Fig. 4. The full-length blots show specific protein bands marked in the figure. Line1: induced group; Line2: uninduced group; Line 3: induced group; Line 4: uninduced group. All Western Blot experiments were repeated three times. [file 12917_2022_3420_MOESM1_ESM.pdf]
